# Pastoralist knowledge of sheep and goat disease and implications for peste des petits ruminants virus control in the Afar Region of Ethiopia

**DOI:** 10.1016/j.prevetmed.2019.104808

**Published:** 2020-01

**Authors:** Bryony Anne Jones, Adem Muhammed, Esmael Tessema Ali, Katherine M. Homewood, Dirk Udo Pfeiffer

**Affiliations:** a Veterinary Epidemiology, Economics and Public Health Group, Department of Pathobiology and Population Sciences, Royal Veterinary College, Hawkshead Campus, North Mymms, Hatfield, AL9 7TA, United Kingdom; b Vétérinaires Sans Frontières Germany, Country Office Addis Ababa, PO Box 2278/1250, Ethiopia; c University College London, Department of Anthropology, 14 Taviton Street, London, WC1H 0BW, United Kingdom; d Department of Infectious Diseases and Public Health, Jockey Club College of Veterinary Medicine and Life Sciences, 5/F, Block 1, To Yuen Building, 31 To Yuen Street, City University of Hong Kong, Tat Chee Avenue, Kowloon, Hong Kong

**Keywords:** Small ruminant, Infectious disease, Disease surveillance, Indigenous knowledge, Disease control, Veterinary anthropology

## Abstract

Pastoralist areas of Ethiopia are vulnerable to drought, causing livelihood loss and famine. One approach to increasing pastoralist resilience is the control of livestock disease, but there is limited information from pastoralist areas to inform control strategies. This study aimed to explore pastoralist concepts of small ruminant disease and implications for infectious disease surveillance and control in the pastoralist Afar Region.

During 2013–14, qualitative and quantitative methods were applied in two villages of one district in the mid-west of the region. Semi-structured group interviews, incorporating participatory tools, explored pastoralist knowledge of small ruminant diseases and their impact. These were followed by multiple visits in different seasons to 70 households for semi-structured and informal interviews, observation of management practices, clinical examinations, and weekly questionnaires of mortality and morbidity. Thematic analysis was applied to interview transcripts and field notes, and descriptive statistical analysis to quantitative data.

Afar concepts of disease causation, terminology and treatment were predominantly naturalistic, related to observable signs and physical causes, rather than personalistic factors (misfortune due to magical or spiritual agents). Disease occurrence was associated with malnutrition and adverse weather, and disease spread with contact between animals during grazing, watering and migration. Disease occurrence varied by season with most syndromes increasing in frequency during the dry season.

Names for disease syndromes were related to the main clinical sign or body part affected; 70 terms were recorded for respiratory syndromes, diarrhoea, sheep and goat pox, lameness, skin diseases, ectoparasites, urinary and neurological syndromes and abortion. Some syndromes with pathognomonic signs could be linked to biomedical diagnoses but most were non-specific with several possible diagnoses. The syndromes causing greatest impact were diarrhoea and respiratory disease, due to mortality, reduced milk production, weight loss, abortion, weak offspring and reduced market value. Afar applied a range of traditional methods and modern medicines to prevent or treat disease, based on livestock keeper knowledge, advice of local specialists and occasionally advice from district veterinarians or animal health workers.

In relation to surveillance for peste des petits ruminants (PPR), several terms were used for PPR-like syndromes, depending on the predominance of respiratory or diarrhoea signs. Therefore, whenever these terms are encountered during surveillance, the associated disease events should be fully investigated and samples collected for laboratory confirmation. The Afar naturalistic concepts of disease parallel biomedical concepts and provide a good foundation for communication between veterinarians and pastoralists in relation to PPR surveillance and control measures.

## 1 Introduction

Pastoralist communities in the arid and semi-arid areas of sub-Saharan Africa face many challenges related to their physical and socio-political environments. The main physical constraints are low and variable rainfall and vegetation growth, which have customarily been managed through mobility and transhumance, common property resource management, and the supplementation of pastoralism with other activities such as crop production, fishing or trading (Homewood, 2008a). Common property resource management is the joint ownership and management by local users of common pool resources. For pastoralists, management of the rangeland by traditional institutions makes movement of livestock possible, by regulating who has access to resources and when they can be accessed, depending on need (Niamir-Fuller and Turner, 1999; Homewood, 2008b). The main social and political constraints, such as land tenure and access, marginalisation and conflict, put restrictions on traditional pastoralist strategies and, combined with physical constraints, lead to increased vulnerability to drought and famine, loss of livelihood and poverty, and migration to urban areas (Homewood, 2008a; Catley et al., 2013). In addition, the regional and global trends of climate change, human population growth and globalisation are also affecting pastoralist areas (Davies and Nori, 2008). While pastoralist systems are more able to adapt to climate variability and uncertainty than other production systems, the effects of climate change and other challenges are pushing them to the limits of adaptation (Davies and Nori, 2008). In Africa, there is already a trend of decreasing rainfall, increasing temperature and increasing incidence of drought (Niamir-Fuller, 2016). Although future predictions are uncertain, there is likely to be an increase in temporal and spatial variability of rainfall and an increased frequency of extreme events causing drought, flooding or temperature extremes, which will have an impact on rangeland productivity, and therefore food security and livelihoods (Herrero et al., 2016). Increasing human populations are causing urban expansion and extension of crop agriculture onto rangelands. Together with large-scale land acquisitions for food or bio-fuel production and land set aside for conservation, pastoralists are increasingly restricted to fragmented and less productive rangelands (Homewood, 2008a; Niamir-Fuller, 2016; Zoomers, 2010). At the same time, increasing pastoralist populations are leading to a higher ratio of people to livestock with access to a decreasing land area (Niamir-Fuller, 2016).

Another major challenge for pastoralism is a high burden of livestock disease causing production losses and mortality that could be mitigated by improved access to animal health services (Niamir-Fuller, 2016; Zinsstag et al., 2016). Drought exacerbates the impact of disease: malnourished animals are more susceptible to infection and less likely to recover from disease. Some of the key characteristics of pastoralism – mobility, communal resource use and social support – increase the risk of pathogen transmission. However, biosecurity measures to reduce risk of disease transmission through movement restriction could radically change these production systems and limit their capacity to adapt to wide variations in rainfall, forage and water availability, making the systems less productive and less resilient.

In relation to improving animal health, animal health services for livestock disease surveillance and control should be strengthened and adapted for mobile pastoralist communities, by combining the experiences, concepts and priorities of pastoralist communities together with empirical data. A better understanding of pastoralist systems will contribute to the development of more appropriate and effective strategies for the control of the most important diseases, and will limit their impact on livelihoods, animal welfare and human well-being, while making more efficient use of scarce resources (Scoones, 1995; Fratkin, 1997; Zinsstag et al., 2016). An important issue when conducting research on animal health in pastoralist systems is the difference in knowledge systems and practices between formally-trained veterinarians who have been educated within the positivist scientific medical paradigm (biomedicine), focussing on the investigation, prevention and treatment of diseases, and the local knowledge of livestock keepers, which prioritises the survival and reproduction of the herd and household, and is derived from traditional knowledge passed down through generations and their own observations and experiences (Waller and Homewood, 1997).

In the 1970s and 1980s, animal health professionals involved in livestock development programmes in developing countries became aware that conventional approaches to the study of animal health were not capturing the complexity of pastoralist systems or the valuable local knowledge of livestock management in marginal areas, and started to collaborate with anthropologists to gain a better understanding of livestock systems. This approach, called “veterinary anthropology” (Sollod and Knight, 1982) or “ethno-veterinary research and development” (McCorkle, 1986), aimed to integrate livestock keeper and researcher knowledge for a holistic understanding of context, problems and potential solutions. This approach has been applied in a variety of livestock production systems across the world including pastoralist, agro-pastoralist and small-holder systems in Africa, Asia, South America and Europe (McCorkle et al., 1996).

Also in the 1980s, participatory rural appraisal (PRA) approaches were being developed to address weaknesses in conventional methods for data collection in rural communities (Chambers, 1992). PRA was adapted and applied by veterinarians in developing countries with the aim of addressing gaps in animal health service delivery in marginalised areas. PRA methods such as community meetings, semi-structured interviews, ranking, mapping and timelines were used to investigate local disease terms, their importance and distribution, disease mitigation practices, and to identify potential solutions to important animal health problems (Kirsopp-Reed, 1994; Leyland, 1994; Waters-Bayer and Bayer, 1994). PRA methods were also applied by veterinarians to specific animal disease issues, such as active clinical surveillance in the final stages of rinderpest eradication, termed “participatory disease searching” (Mariner et al., 2012). Participatory methods were developed for field research on livestock disease problems in pastoralist areas, often complemented by clinical examination and laboratory diagnostics (Catley et al., 2001; Catley and Mariner, 2002). The use of PRA methods for surveillance and field studies in animal health became known as “participatory epidemiology” (PE) (Catley et al., 2012). Semi-structured interviews and PRA tools are now widely used in field studies to explore a variety of animal health issues in developing countries, as part of qualitative studies or in combination with quantitative methods, with variation in the focus of analysis between quantitative and qualitative methods (for example Shiferaw et al., 2010; Bett et al., 2009; Upjohn et al., 2013; Scantlebury et al., 2015).

In this study, we chose to combine qualitative methods to explore local knowledge and understanding of disease together with quantitative methods to quantify disease occurrence, in order to best answer our research question. The qualitative and quantitative methods were implemented in parallel so that each informed the other during the course of data collection and analysis. Our approach was anthropological - prioritising ethnographic methods such as informal interviews and participant observation to document local knowledge of livestock disease with a small number of people and their flocks during repeated visits to the same households and villages over an extended period (15 months), combined with structured and semi-structured interviews and the clinical examination of sick animals (Bernard, 2011). Our analysis of animal health knowledge and systems was informed by theories from medical anthropology of health knowledge and medical systems. Health knowledge has been categorised into two types, naturalistic and personalistic (Foster, 1976; Pool, 1994). A naturalistic health knowledge system is one in which disease is understood to originate from natural forces or conditions such as cold, heat, wind or an upset in the balance of the basic body elements, and treatment focuses on the alleviation or cure of clinical signs. By contrast, in personalistic systems, disease or other misfortunes are caused by the purposeful action of an agent and targeted at an individual. The agent may be human, such as witches or sorcerers, non-human, such as ghosts or evil spirits, or supernatural, such as a god. Treatment takes the form of rituals to counteract these forces (Foster, 1976; Mathias et al., 1996; Green, 1998; Pool and Geissler, 2005). Naturalistic disease knowledge systems incorporating some spiritual components have been reported in pastoralist and agro-pastoralist groups in East Africa (Schwabe and Kuojok, 1981; Heffernan et al., 1996; Waller and Homewood, 1997). In general, animal health knowledge systems are usually a mix of naturalistic and personalistic elements, lying on a continuum between predominantly naturalistic to predominantly personalistic systems (McCorkle and Mathias-Mundy, 1992). A medical system can be defined as a community’s ideas and practices relating to illness and health. There are three main types of system; professional in which biomedicine predominates, traditional which may be predominantly naturalistic or personalistic, and popular or pluralist in which traditional, biomedical and religious medicine are practised side by side (Last, 1981; Pool and Geissler, 2005). In a “popular” medical system, individuals choose who to consult and when and, based on the advice received, decide what action to take, determining for themselves what is effective and when to try something else (Pool and Geissler, 2005).

PPR is a transboundary viral disease of sheep and goats that is endemic in many countries of Africa and Asia, and is a major threat for pastoralist and small-holder farmers, making a significant impact on food security, livelihoods and trade (Banyard et al., 2010). PPR has recently been identified as a target for global eradication (OIE-FAO, 2015), but there are important knowledge gaps in understanding the patterns of occurrence of PPR in extensive production systems where small ruminant movement for access to resources, social support and trade underpin the sustainability of the systems. In Ethiopia, the first clinical suspicion of PPR was in goats in Afar (Pegram and Tereke, 1981, cited by Roeder et al. (1994)) and the virus was later confirmed as the cause of an outbreak in goats from southern Ethiopia (Roeder et al., 1994). In 1999, a national serological survey found that PPR antibody sero-prevalence was highest in the lowland pastoralist Somali and Afar Regions, and the highland Tigray region (Waret-Szkuta et al., 2008). It is hypothesised that the lowland pastoralist areas maintain PPR virus circulation and that there is seasonal spill-over into neighbouring highland populations, but data on disease occurrence in pastoralist areas are sparse. During 2008–2011, as part of emergency interventions in response to drought, approximately 15 million sheep and goats were vaccinated against PPR in pastoralist areas, leading to a reduction in reported outbreaks (MOA, 2013). Ethiopia has developed a national plan to eradicate PPR virus through strengthening surveillance and outbreak detection to allow targeted vaccination for virus elimination (MOA, 2013).

In this context, a research project was developed that aimed to gain a better understanding of the pastoralist production system and patterns of small ruminant disease in the Afar Region of Ethiopia, to support the development of more effective approaches to infectious disease surveillance and control (Jones, 2018). Here we report the findings of one component of that project, which aimed to explore pastoralist concepts of small ruminant disease and implications for PPR surveillance in the Afar Region.

## 2 Materials and methods

### 2.1 The study area

**Fig. 1:**
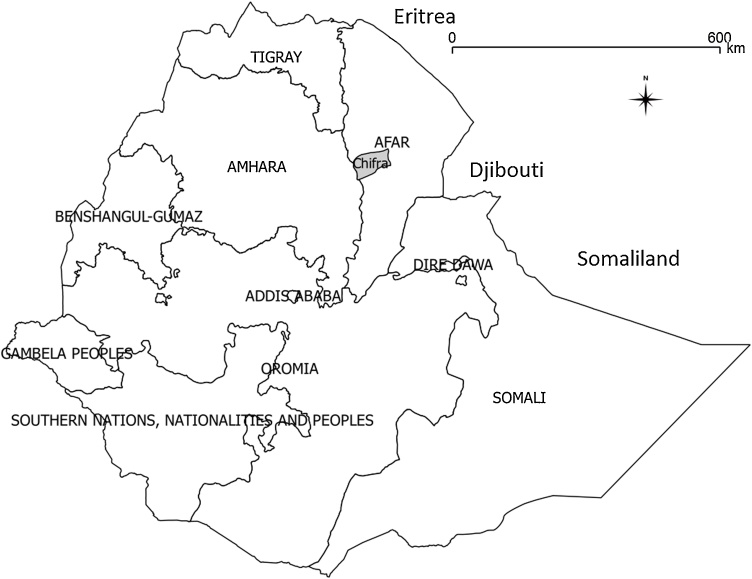
Map of the administrative regions of Ethiopia. Afar Regional State is in the north-east. Chifra district, where the study villages were located, is highlighted in grey in the mid-west of Afar Region.

This study was conducted between October 2013 and December 2014 in Chifra district in the mid-west of Afar Region at the foot of the Rift Valley escarpment on the border with Amhara Region (Fig. 1). The Afar Region is an arid to semi-arid lowland area lying between 9-14ᵒ latitude north and 40-42ᵒ longitude east, with a land area of approximately 95,000 km^2^, bounded to the west by the eastern edge of the Ethiopian highlands (Tigray, Amhara and Oromia Regions), the north by Eritrea, the north-east by Djibouti, and the south by Oromia and Somali Regions. The rainfall pattern is bimodal with a short rainy season during April and a main rainy season during July to August, but rainfall is temporally and spatially highly variable.

The region is populated mainly by the Afar people, numbering approximately 1.4 million (CSA, 2007), who are part of the South-east Cushitic-speaking group together with the Somali, Oromo, Beja and Rendille peoples of eastern Africa (Said, 1997; Homewood, 2008c). The main source of livelihood is mobile multi-species pastoralism, keeping herds of camels, cattle, sheep and goats, and a few donkeys. This is complemented by a variety of other livelihood activities, which vary across the region depending on climate, altitude, vegetation and access to resources, including crop farming, trading, waged labour, salt-mining and charcoal-making (Gebre-Mariam, 1987, 1991; Getachew, 2001; Davies, 2003; SC-UK, 2006; Hassen, 2008). Wide variations in rainfall and forage are managed through herd mobility, fluctuating herd size and traditional mutual support systems (Said, 1997). The size of the livestock population in 2006 was estimated to be 1.9 million cattle, 2.8 million sheep, 2.8 million goats, 0.6 million camels and 95,000 donkeys (BoFED, 2009). The region has faced repeated droughts in the last few decades, causing famine and loss of livelihood (Lautze et al., 2003; Muller-Mahn et al., 2010; Tsegaye et al., 2013).

A preliminary visit was made to the Afar Region in May 2013 by the first and third authors to meet with regional animal health personnel to obtain an overview of the livestock disease situation, and to visit several districts as possible study sites, to gain an understanding of the pastoralist system and to pilot data collection methods. Chifra district was chosen as the study area because it was secure compared to areas further north, and was relatively less researched compared with areas in the south and east of the region. Two pastoralist villages were purposively selected, one from the west and one from the east of the district, using the following criteria; pastoralist, reasonably accessible but not too close to the town (10–40 km away), and not frequently visited by external actors such as researchers and non-governmental organisations (NGOs).

### 2.2 Data collection

The field research team consisted of three people, a British veterinarian and epidemiologist with training in anthropological research methods and field experience in other pastoralist areas of East Africa (first author), an Afar interpreter, and the district veterinary officer (second author, Amharic-speaker) who was assigned by the district authority to accompany the team.

The livestock keepers spoke Afar, and some were able to speak some Amharic. All interviews were conducted in English by the first author with Afar-English interpretation by the interpreter. Some interviews were audio-recorded and later transcribed and translated into English.

The main seasons in Afar are the main rainy season that occurs during July-August (*karma)*, which is followed by the long cool dry season from September to March (*gilal)*. A short rainy season occurs during April (*sugum)*, followed by the hot dry season in May-June (*hagay*). Fieldwork was carried out during four periods between September 2013 and December 2014 with the aim of observing seasonal variation; September-December 2013 (early-mid dry season), February-April 2014 (late dry season & short rains), August 2014 (rainy season) and November 2014 (mid dry season). The preliminary visit was in May 2013 during the hot dry season. The weekly flock dynamics questionnaire was carried out from November 2013 to November 2014.

Initially in each village, a semi-structured interview was conducted with the village leader and a group of 10–15 men and women from the village to introduce the study, to obtain consent to work in the area, and to gain an overview of the characteristics of the village and livestock disease problems, with a focus on sheep and goat diseases. PRA tools were used to visualise information and stimulate discussion; participatory mapping, seasonal calendar and ranking or proportional piling of livestock diseases (Supplementary Information, SI 1). This was followed by a period of about 3 weeks per village during which individual household visits were carried out early in the morning to be able to observe directly the sheep and goat flocks before they left for grazing, and to examine and discuss any clinical cases. A semi-structured household interview was then carried out focusing on management practices, flock structure and dynamics, and disease problems and their impact (SI 1). The types of informant varied between households; in most cases the husband was the main informant with inputs from his wife (or wives) and children, but for some households the wife was the main informant with inputs from her children, her husband being rarely present. Some households in each village were visited multiple times during the 15-month study, to conduct further semi-structured and informal interviews, to follow the progress of the households and flocks over the different seasons, to investigate reports of disease, and to follow up clinical cases. Some of the households migrated during the course of the study and where possible we visited them in their new locations. Additional interviews were carried out opportunistically and as needed to cross-check or gather additional information. A total of 70 households were interviewed during the study period; 23 households in village A and 47 households in village B, of which 13 in village A and 15 in village B were visited multiple times. A woman was the main informant for 7 of the households in village A (30%) and 8 of the households in village B (17%). When visiting flocks, a general examination of the flock was carried out by walking among the animals, and the livestock keepers were encouraged to point out any sick animals for further examination. The clinical examination of a sick animal was a systematic examination from head to tail looking for abnormalities, which included the examination of eyes, nose, mouth, body condition, respiration, mobility and rectal temperature. During clinical examination, an informal interview was carried out to find out the local name for the disease problem, the history including any treatment given, and the characteristics of the disease problem.

After the initial group and household semi-structured interviews, informal interviewing became the main method of data collection when visiting households and opportunistically when walking around the village, looking at flocks, examining sick animals, visiting watering places, when invited to drink milk or coffee or share food at a household, or when sitting in the shade to rest or review data. In response to reports of disease problems, visits were also made to villages in other parts of the district, where group and individual interviews were conducted about the disease situation, common sheep and goat diseases, and clinical examinations carried out.

Based on the initial interviews, a structured questionnaire was developed to obtain weekly quantitative flock dynamics data for 14 flocks in village A over a 12-month period, including the number of animals that died and the cause of death, and the number of animals that were sick and the cause of sickness (SI 2). Data collectors from the village were trained to administer the questionnaire by face-to-face interview. The questionnaire was translated into Amharic script because that was the language that the data collectors could read and write, but they conducted the interviews in Afar language.

Since PPR was a disease of particular interest for the study, when suspected cases of PPR were identified based on the presence of clinical signs (ocular discharge, nasal discharge, mouth lesions, coughing, sneezing, diarrhoea, fever) in a group of animals, conjunctival swabs were collected from two to four animals in the early stages of disease and examined for the presence of PPR antigen by a rapid diagnostic test (Baron et al., 2014). Conjunctival and nasal swabs were collected from the same animals and put into virus transport media in a cool box with ice packs, and then transported to the town where they were stored at −20 °C until the end of the period of fieldwork. The samples were then transported in a cool box with ice packs to the National Animal Health Diagnostic and Investigation Centre (NAHDIC) for diagnostic testing by icELISA (immunocapture enzyme-linked immunosorbent assay) and RT-PCR (reverse transcriptase polymerase chain reaction).

### 2.3 Data management and analysis

Initial group and household semi-structured interviews were audio-recorded, transcribed and translated from Afar into English. During all interviews, observation and clinical examinations, field notes were made. These were reviewed and annotated later the same day, and issues for further enquiry noted. The completed structured questionnaires were checked by the interpreter who translated open text responses into English, and the data were entered into an Excel spreadsheet. Issues were noted for further enquiry during informal interviews.

Interview transcripts and field notes were analysed using NVivo 10 (QSR International http://www.qsrinternational.com). All records were thoroughly read, inductively coded, and a narrative description was developed for the main themes identified (Bernard, 2011; Braun and Clarke, 2006). Quotations from the transcripts that illustrated the themes were identified. The relative frequency of disease occurrence and seasonal variations were estimated by descriptive analysis using Stata IC 12.1 (StataCorp LLC http://www.stata.com).

### 2.4 Ethical approval

Ethical approval for this study was obtained from the Royal Veterinary College Ethics and Welfare Committee and the University College London Department of Anthropology. In Ethiopia, the Ministry of Agriculture Animal Health Directorate provided written approval for the study. The livestock keepers who participated in the study gave their oral consent to participate after the objectives and scope of the project had been explained.

## 3 Results

### 3.1 Naturalistic concept of disease

**Fig. 2:**
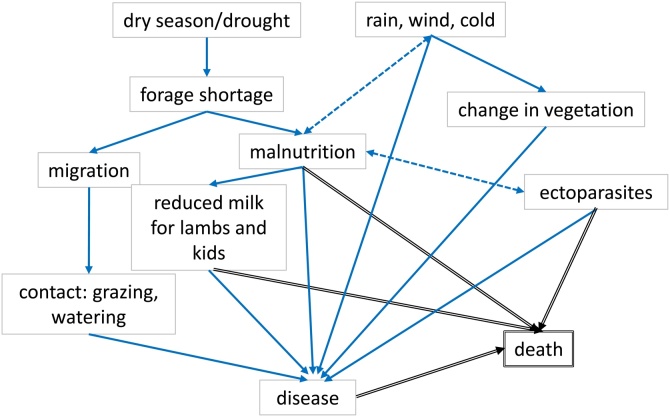
Causal diagram of Afar concepts of factors affecting disease and death. Solid arrows indicate direction of causation. Dashed two-headed arrows indicate interaction between two factors to exacerbate disease. Arrows with double lines indicate that a factor causes death.

The concepts of disease causation, terminology and treatment of the Afar pastoralists in the study villages were predominantly naturalistic, relating to natural rather than supernatural factors, but there was also a spiritual element related to their Islamic religion, with Allah having control over health and outcomes of disease in general. Fig. 2 provides a summary of the factors described as causing or being associated with disease and death of small ruminants and how they are linked or interact with each other. Disease occurrence was associated with malnutrition, adverse weather and certain seasons, ectoparasites and animal movement. Disease in general or specific diseases were attributed to drought:

“If there is drought, there are plenty of diseases. A person who is not hungry [has enough food], does not get sick.” (group interview with women, village A)

“The root cause of the disease [referring to *gublo*] is drought, it happens because of drought and cool weather, especially drought.” (group interview with men and women, village A)

Shortage of forage due to drought forced them to migrate with their herds and flocks, and disease and death of animals occurred due to lack of forage and exposure to diseases during migration. When lactating animals did not have enough forage they did not produce enough milk, and kids and lambs suffered from disease. The long dry season from September to March was associated with most of the common sheep and goat disease problems due to decreasing availability of forage, and ectoparasites were reported to increase towards the end of the dry season debilitating the animals and predisposing them to other diseases.

“*Inkata* [a type of ectoparasite] outbreaks happen whenever drought happens, and consequently diseases like *sura’atu, goson, gublo, undahi* and others attack the animals. The *inkata* sucks all the blood of the animal and finally kills it.” (household interview in village A)

The rain, wind and cooler weather of the main rainy season was associated with certain disease problems; a type of diarrhoea was linked to the change in vegetation after rain, skin disease occurred after heavy rain, and lameness increased in muddy conditions. These ailments were exacerbated by weak body condition after the long dry season. Infection with lice predisposed animals to other diseases, and ticks were reported to be the cause of some skin diseases and lameness.

Disease was described as being spread through contact between sick and healthy animals when they mixed during grazing, watering or when enclosed together:

“Animals may get it [referring to *gublo*] from other sick animals. If there is one infected animal and if it gets mixed up with other healthy animals, they may get it from it. Or if the sick one drinks water with healthy ones or if it slept with healthy animals in the same field.”(group interview with men, village B)

Animals could bring disease from other areas when they were moved for grazing and water resources.

“It [referring to disease] comes from far places via animals. It is communicable. Once the disease finds itself here, it transmits from one animal to the other.” (individual interview, village A)

Some diseases could also be spread by the wind.

“Suppose if there is an outbreak of a disease in Awra [neighbouring district], the wind brings it towards our land. Be it the animal disease or human disease, it comes by the wind.” (individual interview, village A)

### 3.2 Medical pluralism

A range of traditional and biomedical methods were applied by the livestock keepers to prevent or treat disease and to promote health. Traditional treatments included herbal preparations that were administered as a drench, intra-nasally or topically, and substances such as salt, animal fat, butter, honey, kerosene or diesel that were applied topically. Fractures were corrected by splinting, and manual correction of dystocia and fetotomy was practised. Preventive measures included cleaning of enclosures, periodic relocation of the household compound, separation of sick animals from healthy, feeding minerals through salty water sources, salty grazing or feeding of rock salt, as well as grazing management to optimise nutrition. Most of the traditional practices were naturalistic, aiming to prevent or cure a physical cause or sign, but there were also communal prayers to Allah for the good health of people and animals.

Access to formal veterinary services was very limited at village level; although village animal health posts had been built they were not manned. In the main town, there were district veterinarians and animal health assistants, but they had limited resources and rarely visited the villages, so livestock keepers purchased basic medicines from the town market or the veterinary pharmacy and occasionally sought advice from veterinary personnel. Vaccination campaigns against PPR, sheep and goat pox and pasteurellosis were carried out sporadically by the Regional Animal Health Department and NGOs, when vaccine and funds for vaccine delivery were available. People were familiar with the vaccination of children and livestock to prevent disease, and were willing to have their animals vaccinated but had little control over when and where it was carried out, and which diseases were targeted.

If an animal became sick, the livestock keepers could decide to treat the animal themselves using traditional methods or biomedical methods or both, based on their own knowledge or the advice of family members or friends. Knowledge of diseases was variable within the villages; most people knew the more common diseases, but some individual men and women showed more interest and provided more detailed descriptions and explanations of diseases and how to prevent and treat them. Certain individuals were considered to be specialists in treating certain conditions, such as a local leader who had expertise in dealing with dystocia (difficulty giving birth).

### 3.3 Local disease terms

The Afar language terms for livestock diseases were mainly related to a major clinical sign or the main body part affected. A total of 70 terms were recorded for respiratory diseases, diarrhoea, sheep and goat pox, lameness, skin diseases, ectoparasites, urinary and neurological syndromes, and abortion. These are listed alphabetically for reference as supplementary information (SI 3). The most frequently mentioned terms are described below, and more detailed descriptions are provided as supplementary information (SI 4, SI 5). The terms have been grouped by the body system affected, which reflects the way that the livestock keepers talked about their disease problems. In group and household interviews, the livestock keepers described one or more respiratory syndromes, diarrhoea syndromes, skin problems, ectoparasites and lameness. Less frequently, reproductive, urinary or neurological problems were described. A few terms used for syndromes with pathognomonic signs could be linked to biomedical diagnoses, but most terms were used for syndromes that had several possible diagnoses. Some disease terms were widely used and consistently described and applied within the two villages and in other parts of the district and region, and clinical cases were consistently observed that fitted these descriptions. For example the terms, *gublo* (lungs) was widely used for a respiratory disease and *uruga* (diarrhoea) for diarrhoeal disease. Some syndromes had more than one name, for example the term *korboda* (stones on neck) was used for a pox-like syndrome in one village while the same syndrome was called *waybo* (no literal translation obtained) was used for the same syndrome in the second village, although people from both villages understood both terms and said that *korboda* was the same as *waybo*. When examining clinical cases, different terms might be used by different people to describe the same case, for example, an animal with skin disease was described by one person as *sandera* and as *hamma* by another person.

#### 3.3.1 Respiratory syndromes

Commonly used terms for respiratory clinical signs were *sura’atu*, *sura’ale* and *sanak*, meaning “nasal discharge”, and *goson*, *kaho* or *kahoenta*, meaning “coughing”. Some of these terms were also applied to respiratory syndromes; *sura’atu*, *sura’ale*, and *goson*. *Gublo* and *mesengele* were Afar words for “lung” and both were used to describe a disease syndrome affecting the lungs. *Furoda* was a disease syndrome that affected the eyes and lungs. The terms were sometimes combined to name a syndrome; *sura’atu-goson* (nasal discharge-coughing), *sura’ale-goson* (nasal discharge-coughing), *sura-gublo* (nasal discharge-lungs), or *goson-gublo* (coughing-lungs). *Sura’atu, goson, gublo* and *uruga* (diarrhoea) could occur together, especially in young animals. One respiratory syndrome could progress into another; *sura’atu* or *goson* could develop into *sura-gublo; goson* could develop into *sura’atu* and then *mesengele*; or *mesengele* could become *goson*.

**Fig. 3:**
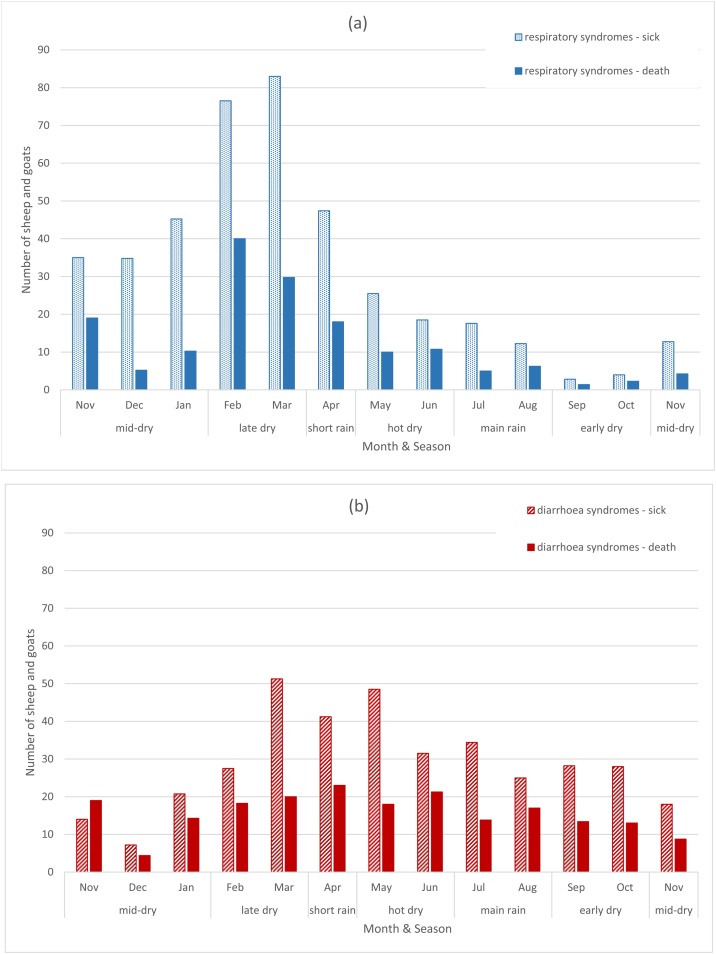
Seasonal distribution of disease and deaths due to a) respiratory syndromes and b) diarrhoea syndromes. The average number of sheep and goats that died or were sick per week as shown for each month of the flock survey (Nov 2013 to Nov 2014). It should be noted that an animal that was recorded as sick in one week and dead in the following week would be included in both datasets.

Respiratory syndromes were reported to occur during the drier and cooler weather in the long dry season, and during drought. This seasonality was supported by the results of the flock dynamics survey. Fig. 3a shows the weekly average number of sheep and goats that died or were sick with respiratory syndromes for each month of the survey. The number of animals affected by respiratory disease increased during the mid-dry season (November to January) to peak in late dry season (February-March).

*Sura’atu* was the most frequent cause of sickness (35.1% of reported sick animals) and the second most frequent cause of death (27.3% reported deaths). Clinical cases of *sura’atu* were commonly seen in both villages with signs of watery, mucoid or purulent nasal discharge, with or without coughing, lacrimation, dyspnoea and weight loss.

#### 3.3.2 Abdominal syndromes

The most common abdominal problem was diarrhoea. The Afar word for diarrhoea was *uruga*, which was used to describe the clinical sign of diarrhoea, and was the name of a disease syndrome for which acute or chronic diarrhoea was the main sign, together with variable signs of nasal discharge and lacrimation. Another diarrhoea syndrome was *undahi* (meaning “slowly”), with signs of blackish or bloody diarrhoea, weight-loss and death. *Bogo biyakita* (sick stomach) was occasionally used for animals with diarrhoea or abdominal discomfort. An animal with *arbite* (meaning “bloated”) developed a swollen abdomen after eating bread or new grass after rain. Fig. 3b shows the weekly average number of sheep and goats that died or were sick with abdominal syndromes for each month of the flock dynamics survey. Cases occurred throughout the year with a decrease in mid-dry season (December) and an increase in the late dry season through the short rains and into the hot dry season (March-May). *Uruga* was the most common cause of death (34.2%) and the second most common cause of sickness (31.4%), while *undahi* caused 7.4% of deaths and 7.7% sickness. Clinical cases of *uruga* were commonly seen during the study, especially during *karma,* but only one case of *undahi* was seen.

#### 3.3.3 Pox syndromes

Two terms, *korboda* (stones on neck) and *waybo* (no literal meaning obtained), were used interchangeably for a disease syndrome that was characterised by the typical skin lesions of sheep and goat pox, which was observed in both villages during the study. Two types of *korboda* were described that occurred at the same time within a flock; external and internal.

“It is a disease that attacks the body. For some, it attacks the external parts of their body and kills them. For some it attacks the internal part of their body. Internally it becomes like *goson*. It discharges diarrhoea.” (Household interview, Village A)

External *korboda* showed small swellings (*duduba*) in the skin all over the body from which most animals recovered, while the signs of internal *korboda* were *karo* (meaning “unwell”) with variable signs such as fever, nasal discharge, mouth lesions, coughing, abdominal pain or diarrhoea.

#### 3.3.4 Skin disease

Several terms were used to describe disease syndromes causing skin lesions. *Agara* (meaning “itching”) was a generalised pruritic condition, while *sandera* and *hamma* (no literal meaning for these terms was obtained) were more localised, with lesions usually occurring on the head, legs and genital areas. The term *dalela* (meaning “wounds”) was used for bite wounds and the lesions of *sandera* and *agara*. The terms *dalela* and *afu-delay* (*afu* means “mouth”) were also applied to a syndrome of lesions around the mouths of young goats. *Duduba* (meaning “swelling”) was used to describe skin abscesses and lumps, such as those associated with *korboda*, infected bite wounds and submandibular or generalised oedema. The term *do’u* (meaning “lump”) was also used to describe more discrete abscesses or lumps in the skin.

#### 3.3.5 Ectoparasites

There were several terms for ectoparasites; *inkata* (meaning “insects”) was the name given to small “insects” that lived in the hair (observed to be lice), while *iba’adu, kilimi* and *silimi* were terms for different types of ticks.

#### 3.3.6 Lameness

Lameness, called *iba kosinta* (meaning “lameness”), *iba* (meaning “leg”) or *kos* (meaning “limp”), was a common problem, with swelling of the interdigital space and above the hoof, associated with ticks and the rainy season. Another lameness syndrome that was described but not observed during the study was *abeb.* Affected sheep and goats suddenly became lame in all four legs, were “sick in the stomach”, and developed wounds in the mouth. Biomedically, these signs are pathognomonic for foot-and-mouth disease (FMD). *Abeb* was also reported to be an important disease of cattle, providing further evidence that this syndrome was likely to be FMD because it affects cattle, sheep and goats.

#### 3.3.7 Reproductive problems

The term *fanache dalte* (meaning “early birth”) was used for animals that had aborted, and was mentioned as a clinical sign of some diseases. Clusters of abortions were observed in several flocks in both villages during the study. Blood samples from five recently aborted animals from three flocks were submitted to the Regional Veterinary Laboratory, and all were strongly positive for brucellosis antibody by the rose Bengal test. From the weekly flock survey results, the estimated annual abortion rate was 4.6% of breeding females in goats and 3.3% in sheep.

### 3.4 Impact of disease

Ranking and proportional piling exercises during group interviews indicated that the disease syndromes causing greatest impact were diarrhoea (*uruga, undahi*) and respiratory disease (*sura’atu, sura-gublo, gublo, goson*), followed by pox (*korboda*), lameness (*iba*) and skin disease (*sandera, agara*). This was supported by the findings of household interviews and clinical examinations. The main criteria used by livestock keepers to indicate impact were mortality, reduced milk production due to death of lactating animals or decreased production from affected animals, reproductive loss due to death of breeding animals, abortions, stillbirths and neonatal deaths, and reduced market value due to poor body condition or skin lesions. The results of the weekly flock survey supported these qualitative findings. The most common cause of death was *uruga* (diarrhoea, 34.2% of reported deaths) followed by *sura’atu* (nasal discharge, 27.3%), while the most common cause of sickness was *sura’atu* (35.1% of reported sick animals) followed by *uruga* (31.4%). Other frequently reported diseases were *undahi* (slowly, 7.4% deaths, 7.7% sickness), *korboda* (stones on neck, 6.0% deaths, 7.8% sickness), *ululu* (meaning “starvation”, 5.9% deaths, 1.0% sickness) and *iba* (lameness, 3.5% deaths, 6.3% sickness). *Sandera* was the most commonly reported skin disease (0.7% deaths, 1.2% sickness). Sheep were more severely affected by all disease syndromes compared to goats, except the skin disease (*sandera*) which more commonly affected goats. Of the clinical cases examined in both villages, respiratory syndromes such as *furoda, goson* and *sura’atu* made up the greatest proportion; 52% of cases in village A and 35% in village B. Other common clinical syndromes seen were *uruga* (diarrhoea)*, waybo* (pox) and *iba* (lameness). *Sandera* was the most common skin disease seen in both villages, and *inkata* (lice) was the most common ectoparasite. Cases of *afu-delay* (mouth wounds) were common in village B but less so in village A.

### 3.5 Peste des petits ruminants (PPR)

Among the disease syndromes described and observed during the study, several had clinical signs that were similar to those of PPR disease, as described in veterinary textbooks and information manuals (Roeder and Obi, 1999; Rossiter, 2004). However, none of the suspected cases were confirmed as PPR by either rapid diagnostic test conducted in the field or icELISA and RT-PCR conducted by NAHDIC. An outbreak that occurred in a neighbouring district after the end of the study period was confirmed as PPR by rapid diagnostic test. The clinical signs observed were nasal and ocular discharge, dyspnoea, diarrhoea and fever, and the flock owner called the disease *gublo* (“lungs”), which is one of the respiratory syndromes described above. During key informant interviews with veterinarians and para-professional animal health personnel (Animal Health Assistants, Animal Health Technicians, Community-based Animal Health Workers) working within the study area and in other parts of Afar Region, it was found that they attributed a variety of local names to PPR disease, and that the local names varied depending on whether the predominant clinical signs were respiratory (*sura’ale, gublo, mesengele*) or diarrhoea (*uruga, abel-uruga -* blood-diarrhoea). Other terms attributed to PPR within Chifra district were *aranwagit* (looking to sky), *korboda* (stones on neck), *undahi* (slowly) and *fododa* (no literal meaning obtained). An animal health worker in the south of Afar said that the name for PPR was *ndugulu*, meaning “drowsy” to describe the drooping ears, lowered head and sleepy eyes of the affected animal. This was a term that was formerly used for rinderpest, a disease of cattle that has been eradicated, caused by a virus related to PPRV and causing similar clinical signs. Also in the same area, a veterinarian and another animal health worker said the term for PPR was *gesohabe*, meaning “the pen (*geso*) is taken away”, which parallels another local term for rinderpest in cattle; *dugahabe* meaning “the village is taken away”. The term *dugahabe* was also used in Chifra for rinderpest but this term was not used for small ruminant disease. It should be highlighted that outbreaks of PPR that had been reported in the region had rarely been laboratory-confirmed so it was uncertain whether the outbreaks being described by the animal health personnel were caused by PPR virus.

Table 1 shows the PPR clinical signs, as described in the veterinary literature, that were reported and/or observed to occur as part of the most common local disease syndromes. This demonstrates that, based on clinical examination, several of the common local disease syndromes would be identified as suspected cases of PPR disease, and laboratory diagnostic tests would be required to confirm or exclude this diagnosis.

**Table 1:** Afar small ruminant disease terms that are associated with PPR clinical signs.

The main clinical signs of PPR (as described in veterinary literature) are listed in the left-hand column, and common local disease syndromes are listed across the top of the matrix. For each local syndrome, the shaded cells indicate which of the PPR clinical signs were reported by livestock keepers to be associated with the syndrome and/or were observed by the researchers when examining clinical cases of the syndrome, as named by the livestock keepers.

## 4 Discussion

The main small ruminant disease syndromes affecting the villages and causing the greatest impact in terms of mortality and loss of productivity were respiratory disease and diarrhoeal disease, followed by sheep and goat pox, lameness and skin disease. Mortality due to disease was highest during mid and late dry season, and sheep were more severely affected than goats.

The pastoralists used a range of traditional and modern medicines and practices. In medical anthropology this has been called a “pluralist” or “popular” medical culture, in which people choose who to consult, what action to take, and decide for themselves what is most effective (Last, 1981; Pool and Geissler, 2005). Pluralist animal health systems have been reported in other pastoralist systems in East Africa. For the Maasai and Samburu, a pragmatic mixture of traditional and modern medicines has been observed with modern medicines and vaccines increasingly being used where experience demonstrated a contribution to herd survival, and due to increasing access to modern medicines (Heffernan et al., 1996; Waller and Homewood, 1997). A similar situation was found for the Dinka in South Sudan (Schwabe and Kuojok, 1981), and in South Africa, where traditional medicines were considered to be effective for some diseases and modern medicines for others (Beinart and Brown, 2013). This flexible, pragmatic approach makes the best use of the resources available to the pastoralists and demonstrates their adaptability, contributing to the resilience of the livelihood system. It also provides an opportunity for strengthening disease control systems: additional effective biomedical disease control measures, such as vaccination and ectoparasite control, were welcomed by the livestock keepers in this study. However, these interventions need to be planned together with the pastoralists to ensure that they are integrated into the daily and seasonal activities that are the foundation of productive sheep and goat rearing in arid and semi-arid rangelands. More regular and effective consultation with livestock keepers to prioritise and plan interventions would increase the impact of existing animal health services. The importance of livestock keeper participation in livestock disease control programmes was exemplified in the efforts to eradicate rinderpest from pastoralist livestock systems in East Africa (Mariner et al., 2012) and will be equally important in the PPR eradication programme (Mariner et al., 2016).

Afar concepts of disease in the study area were predominantly naturalistic, which, when viewed from a biomedical perspective, appears to be rational and corresponds well with scientific theory. External stresses such as adverse weather and poor nutrition are exacerbated by the effect of parasites, leading to greater susceptibility to a variety of pathogens that are spread directly through contact or indirectly through the environment. The parallels between Afar and biomedical concepts provide common ground for communication between livestock keepers and veterinary personnel and the development of interventions to mitigate disease that fit with local disease understandings and can be explained in local terms. Green (1998) asserts that, in general, infectious diseases of humans and animals in African cultures are understood naturalistically, and the concept of disease transmission through contact has also been observed in other African cultures and correlates well with biomedical concepts of contagious disease (Green, 1998). Predominantly naturalistic animal health knowledge systems have been described in other pastoralist and agro-pastoralist communities in East Africa, such as the Issa Somali pastoralists (Gebreyesus et al., 2014), the Nilotic Dinka and Nuer in South Sudan (Schwabe and Kuojok, 1981; Adolph et al., 1996), Samburu in northern Kenya (Heffernan et al., 1996), and Maasai in southern Kenya and northern Tanzania (Waller and Homewood, 1997). For the Nilotic groups, there was also a personalistic element; some disease syndromes were associated with god, usually those characterised by sudden death or those with few clinical or post mortem signs (Adolph et al., 1996), or new diseases suddenly occurring in an area (Heffernan et al., 1996). The Nuer, Dinka and Samburu also recognised that some diseases were contagious and practised isolation of sick animals and quarantine of affected herds (Evans-Pritchard, 1940; Adolph et al., 1996; Heffernan et al., 1996; Majok and Schwabe, 1996). In West Africa, Bonfiglioli et al. (1996) reported that the FulBe (Fulani) in northern Senegal described some diseases to be contagious, being spread through contact between sick and healthy animals, from wild animals, birds and insects, the wind, or by handling a sick animal and then a healthy animal. They took action to limit spread by isolation and quarantine. Other diseases were associated with vegetation, water or soil, seasonal change, or malnutrition, but some individual animals or herds were fated to be inherently vulnerable, and otherwise unexplainable animal health problems were attributed to sorcery. In South Africa, Beinart and Brown (2013) explored local animal health knowledge in several rural areas and found that the farmers’ knowledge of disease causation was mainly naturalistic, associated with pasture, water, nutrition, weather and the seasons, but there was limited awareness of contagious spread. A few diseases in each area were ascribed to witchcraft, the supernatural or offending the ancestors, usually those related to sudden death or diseases affecting only the animals of a single person.

Afar disease terminology in the study area was primarily syndrome-based, describing the main clinical signs or body parts affected. Some disease terms with pathognomonic clinical signs mapped closely to biomedical disease terms, but most terms were non-specific, representing a set of clinical signs that corresponded to several different biomedical diagnoses. There was variation in the use of terms within and between villages, and between different parts of the region, which might be attributed to dialect or variation in disease occurrence by area. At the individual level, variation in use of terms could be related to a person’s direct experience of a disease, combined with what they had learnt from relatives and friends, and possibly their interaction with human health and animal health workers (Grandin and Young, 1996). From a biomedical point of view, respiratory, diarrhoeal or skin diseases have many possible aetiologies, and the clinical signs caused by a specific pathogen can vary depending on the stage of the disease, the host’s immune status, species, age, co-infections and other factors.

There have been a limited number of other studies of local veterinary knowledge in other parts of the Afar Region. In the 1990s, as part of community-based animal health projects there were several unpublished studies that aimed to document traditional Afar knowledge in relation to common livestock disease syndromes using semi-structured interviews with livestock keepers in several sites in central and south Afar (Mariner et al., 1994; PARC, 1994). In 1997, Dagnatchew (2001) conducted a study of indigenous veterinary knowledge and practices in the northwest of Afar using questionnaires and group discussions with livestock keepers and animal health personnel, and observation in the market and villages. More recently, local knowledge of sheep and goat diseases was explored through group interviews with livestock keepers and animal health personnel in four districts of south and central Afar (Gari et al., 2015). These studies described a similar range of disease syndromes, and some of the disease terms recorded were the same or similar to those recorded during this study but there were some major differences between the north, centre and south of the region that are most likely due to variations in dialect across the region. More detail is provided in the supplementary information (SI 5). One of the limitations of some of these Afar studies, and other studies of local veterinary knowledge, has been the use of a single semi-structured group interview to represent an area during which a consensus list of terms and disease descriptions is obtained that implies a single body of consistent knowledge rather variation in knowledge at different scales (Last, 1981; Moritz et al., 2013). We found that using semi-structured interviews followed by clinical examinations combined with informal interviewing promoted more informative discussions than using semi-structured interviews only. Bryman (2004) highlights the importance of participant observation and continued presence for accessing information that is taken for granted and therefore is less likely to come out during an interview. Based on their experiences of exploring ethno-veterinary knowledge of FulBe and Arab pastoralists in Cameroon, Moritz et al. (2013) comment that much local knowledge is practical and best studied through participant observation combined with interviews, rather than in the abstract solely through interview. We argue that larger scale rapid approaches should be complemented by longer term in-depth studies to improve the validity of the information. However, in general veterinarians do not receive any training in qualitative research methods during under- or post-graduate courses, whether in developed or developing countries, and there tends to be a lack of understanding and respect for knowledge systems that differ from the biomedical paradigm. Greater awareness of other ways of knowing would provide a foundation for improved communication between veterinarians and livestock keepers, and more appropriate animal health service delivery. As part of their training in research methods, veterinarians should be introduced to qualitative as well as quantitative approaches. The promotion of “participatory epidemiology” was an important step in this direction, but training in PE has generally been a short practical course in the use of semi-structured interviews and PRA tools, with little time spent on the epistemological principles of qualitative approaches, and therefore the PE approach falls short because it tends to be used for rapid information-gathering, without taking time for observation and informal interviews, and lacks the rigour of qualitative analysis.

The variation in specificity of local disease terms in relation to biomedical terms and the problem of equating local disease terms based on observable clinical signs with biomedical disease names based on aetiology has been highlighted by other authors and can lead to inappropriate treatment advice to livestock keepers (Sollod et al., 1984; McCorkle, 1986; Beinart and Brown, 2013). Grandin and Young (1996) discussed the diversity of disease names used by various ethnic groups in East Africa and observed that a local disease term may refer to several biomedical diseases that have similar signs, and one (biomedical) disease may have more than one local disease name due to different signs at different stages of the disease or in different age groups. Heffernan et al. (1996) explored ethno-veterinary knowledge with Samburu agro-pastoralists in northern Kenya and found that there were different local disease terms for different clinical presentations of the same (biomedical) disease. Adolph et al. (1996) conducted a study of ethno-veterinary knowledge in agro-pastoralist Dinka and Nuer communities in South Sudan and found that some disease terms were names for syndromes that corresponded closely with biomedical diseases, some had names based on a clinical sign but represented a syndrome with a wider set of signs, and some local diseases changed to another local disease as the clinical signs of a (biomedical) disease progressed. They cautioned against pairing local and biomedical terms. Catley et al. (2001) investigated a chronic weight loss syndrome in cattle in South Sudan, which was generally assumed to be trypanosomiasis, and found that most affected animals had a mixed infection of trypanosomiasis, liver fluke, parasitic gastro-enteritis and schistosomiasis. In their study of local animal health knowledge in South Africa, Beinart and Brown (2013) suggested that some direct translation from local to English terms was justified, but there were non-specific terms that related to more than one biomedical disease, and there was diversity in naming the same set of signs within and between sites. It is important therefore that veterinary personnel are encouraged (and have the resources) to spend time with livestock keepers and their flocks to understand local disease terms and clinical syndromes and their variability. They should be aware that local disease terms relate to syndromes and not to specific biomedical diagnoses, except where there are obvious pathognomonic signs that are recognised by both veterinary personnel and pastoralists.

In relation to surveillance for PPR as part of an elimination programme, it will be important to have a sensitive surveillance system with multiple components including livestock keeper disease reporting and active disease searching. The Afar livestock keepers in this study did not appear to use a specific term for PPR disease, which is not unexpected given the syndrome-based disease terminology and the variability of clinical syndromes that can occur with PPR disease, ranging from mild, mainly upper respiratory tract signs, to pneumonia and/or diarrhoea. When PPR is first introduced into a naïve population there is usually a high mortality epidemic with severe distinctive clinical signs (Roeder et al., 1994), but when the virus has been present in an area for some time, there is reduced mortality and severity of signs, making it less easy for veterinarians and animal health personnel to distinguish it from diseases with similar signs. Veterinary personnel involved in PPR surveillance in this area should be aware that there is no single local disease term for PPR disease, and several of the commonly occurring local disease syndromes have some or most of the clinical signs of PPR. This should be taken into account when conducting passive and active surveillance: PPR should be considered as a differential diagnosis of local disease terms for respiratory, diarrhoea, stomatitis and ocular/nasal discharge syndromes (Table 1). During a clinical investigation, some syndromes can be rapidly excluded based on the presence of pathognomonic signs, such as the skin lesions of external *korboda,* which are typical of sheep and goat pox. However, most syndromes will require full history-taking, clinical examination and confirmation of a PPR diagnosis by rapid field diagnostic test or laboratory diagnostic test to confirm or exclude the diagnosis. More routine use of diagnostic tests will lead to a better understanding of the sensitivity and specificity of local disease terms in relation to laboratory-confirmed PPR i.e. which terms are most likely to be associated with true PPR disease. Based on the findings of this study, it is likely that a number of suspected PPR outbreaks may not be caused by PPR virus but may be other diseases that share similar clinical signs. It is therefore very important that an outbreak is fully investigated and confirmed as PPR before starting vaccination because the vaccination will not be effective at controlling the outbreak if the disease is not PPR, and will lead to loss of livestock keeper confidence in vaccination.

The combination of qualitative and quantitative methods used in this study generated rich information on local disease terminology and occurrence in the study villages, but the purposive selection of villages and a non-random sample of households means that care should be taken when drawing generalised inferences or extrapolating the study results to other populations. However, the results provide an example that is likely to be of relevance to similar contexts in the Afar Region and in other pastoralist areas of eastern Africa. They contribute to an improved understanding of small ruminant disease in a marginalised pastoralist area to support more appropriate and effective disease surveillance and control strategies. Some of the production losses due to diseases are easily preventable through use of safe and effective vaccines. Vaccination campaigns that are planned together with the livestock keepers and coordinated across the region and with neighbouring regions, could eliminate a disease such as PPR from the region, leading to improved productivity, food security and more resilient livelihoods.

## Funding

This work was supported by the Royal Veterinary College Research Office and the Biotechnology and Biological Sciences Research Council (BBSRC) Animal Health and Welfare ERA-Net Project: Improved Understanding of the Epidemiology of Peste des Petits Ruminants (IUEPPR) [grant number BB/L013592/1].
