# Supplementary material for: Pastoralist knowledge of sheep and goat disease and implications for peste des petits ruminants virus control in the Afar Region of Ethiopia
Source: Prev Vet Med. 2020 Jan;174:104808. doi: 10.1016/j.prevetmed.2019.104808 (PMC6983938; doi:10.1016/j.prevetmed.2019.104808)
Supplement: Supplementary file 1 [file mmc1.docx]

### Supplementary Information 1. Semi-structured interview guides and participatory tools

### 1 Introductory meeting with *Kebele* leader

- purpose of study
- basic information on kebele – human population, villages, key resources (mapping of kebele), livestock population
- current situation in kebele – water, grazing, animal health, wildlife
- identification of key informants – CAHWs, extension officers, traditional healers, other
- selection of study village/villages, introduction to village leader

### 2 Introductory meeting with Village leader and elders, in each village

- purpose of study
- basic information on village – human population, key resources (mapping of village and resources), livestock population, wildlife
- current situation in village – water, grazing, animal health
- main sheep and goat diseases – names, descriptions (ranking, proportional piling)

### 3 Household interviews

- flock management
  - daily management routine, seasonal variations, household roles
  - movements – water, grazing, daily, seasonal
  - contact with neighbouring villages/flocks
- flock structure - proportional piling
- flock dynamics – births, deaths, incoming (gifts, purchases, other), outgoing (gifts, sales, other), progeny history
- marketing
- main diseases of sheep and goats
- local name, clinical and post mortem signs, age groups affected, species affected, aetiology (ranking, proportional piling)
- action taken to prevent, treat or control diseases

### 4 Participatory tools

Some participatory tools were used during semi-structured interviews to explore particular issues or to stimulate discussion. The methods were adapted from those used during participatory disease surveillance (Mariner and Paskin, 2000; Catley, 2005; Letereuwa et al., 2007; Ameri et al., 2009).

##### Participatory mapping

Participatory mapping was used during introductory group interviews to each village and its key features such as grazing and watering places and migration of flocks. The maps were prepared on A2 flip chart paper with marker pens. For orientation, the current location was marked in the centre of the paper together with a key feature such as a building, road or mountain. The participants were then asked to draw or indicate the locations of the features of interest. This provided a basis for further discussions about resources and movements.

##### Seasonal calendar

A seasonal calendar was constructed during a group interview in village B. Having clarified the local terms for seasons, the seasons were written across the top of a matrix on an A2 flip chart paper. In the left column of the matrix, the variable rainfall was written in the first row. The participants were given 30 counters and asked to divide the counters between the seasons to indicate the relative amount of rainfall by season. This was repeated for other variables related to the rangeland, livestock production and diseases. The results provided a basis for further discussion of seasonal events with the participants.

##### Simple ranking

Simple ranking was used during the village B group interview to rank livestock species by number, and by livelihood importance, and was used in several group interviews to rank small ruminant diseases by importance. After discussion of the livestock species kept, or the common diseases present, the names of species or diseases were written on individual cards, and spread on the ground. The participants were asked to rank the cards based on a criteria (number, importance), and the reasons for the ranking.

##### Proportional piling

Proportional piling was used in some group and household interviews to explore relative morbidity and mortality of the main small ruminant diseases. The participants were given 100 beans to represent the whole flock and asked to show the proportion that became sick over a one-year period, and the proportion that stayed healthy. Focusing on the group that became sick, they were asked to show what proportion suffered from each of the main diseases, and other diseases. Out of these groups, they were asked to show the proportion that had died.

##### Progeny history

Progeny history, a method that has been successfully applied for cattle (Homewood et al., 2006), was used during flock examinations with a few households. The woman looking after the flock was asked to identify one of her best female sheep or goats, and then list all its offspring and what had happened to them. This was repeated with one or more other animals. The women did not seem very comfortable with this exercise, expressed as embarrassed laughter and reluctance to respond, so it was only carried out with a few households.

### References

Ameri, A.A., Hendrickx, S., Jones, B., Mariner, J., Mehta, P., Pissang, C., 2009. Introduction to participatory epidemiology and its application to highly pathogenic avian influenza participatory disease surveillance: a manual for participatory disease surveillance practitioners. ILRI, Nairobi, 56.

Catley, A., 2005. Participatory Epidemiology: A Guide for Trainers. African Union Interafrican Bureau for Animal Resources, Nairobi, 116.

Homewood, K., Trench, P., Randall, S., Lynen, G., Bishop, B., 2006. Livestock health and socio-economic impacts of a veterinary intervention in Maasailand: Infection-and-treatment vaccine against East Coast fever. Agricultural Systems 89, 248-271.

Letereuwa, S., Mogga, N., Araba, A., Jones, B.A., 2007. Rinderpest Particiaptory Disease Searching: a manual for veterinarians and animal health workers in Southern Sudan. VSF Belgium, Nairobi, 33.

Mariner, J.C., Paskin, R., 2000. Manual on Participatory Epidemiology: methods for the collection of action-oriented epidemiological surveillance. FAO Animal Health Manual 10. Food and Agriculture Organization of the United Nations Rome.
