# Supplementary material for: Pastoralist knowledge of sheep and goat disease and implications for peste des petits ruminants virus control in the Afar Region of Ethiopia
Source: Prev Vet Med. 2020 Jan;174:104808. doi: 10.1016/j.prevetmed.2019.104808 (PMC6983938; doi:10.1016/j.prevetmed.2019.104808)
Supplement: Supplementary file 2 [file mmc2.pdf]

Code: ኮድ፡

Flock questionnaire

የበግና፡ የፍየሎች፡ መንጋ፡ መጠይቅ

Name of owner የባለቤት፡ ስም		Date of interview የመጠይቅ፡ ቀን	
Village መንደር		Kebele ቀበሌ	

1. During the last one week, how many sheep and goats were born? ባለፈው፡ አንድ፡ ሳምንት፡ ውስጥ፡ ምን ያህል በጎችና፡ ፍየሎች፡ ተወለዱ?			
Male sheep ወንድ፡ በግ		Male goats ወንድ፡ ፍየል	
Female sheep ሴት፡ በግ		Female goats ሴት፡ ፍየል፡	

2. During the last one week, how many sheep and goats did you sell? ባለፈው፡ አንድ፡ ሳምንት፡ ውስጥ ምን ያህል በጎችና፡ ፍየሎች፡ ተሸጡ?			
Lagaday ፡ ለጋዴይ		Moyu ሞዩ	
Anayee አናዬ		Bokole ቦኮሌ	
Gahsuru ጋሹሩ		Giregire ጊሬጊሬ	
Sebeni ሰበኒ		Rehido ሬሂዶ	
Marua ማሩዋ		Debela ደበላ	
ida ኢዳ		Reita ሬይታ	

3. During the last one week, how many sheep and goats did you buy? ባለፈው፡ አንድ፡ ሳምንት፡ ውስጥ ምን ያህል በጎችና፡ ፍየሎች፡ ተገዙ?			
Lagaday ለጋዴይ		Moyu ሞዩ	
Anayee አናዬ		Bokole ቦኮሌ	
Gahsuru ጋሹሩ		Giregire ጊሬጊሬ	
Sebeni ሰበኒ		Rehido ሬሂዶ	
Marua ማሩዋ		Debela ደበላ	
ida ኢዳ		Reita ሬይታ	

4. During the last one week, how many sheep and goats died? (give the number and cause of death) ባለፈው፡ አንድ፡ ሳምንት፡ ውስጥ፡ ምን ያህል፡ በጎችና፡ ፍየሎች፡ ሞቱ? ቁጥራቸውን፡ የሞቱበትን፡ ምክንያት፡ ይግለፁ?			
Lagaday ለጋዴይ		Moyu ሞዩ	
Anayee አናዬ		Bokole ቦኮሌ	
Gahsuru ጋሹሩ		Giregire ጊሬጊሬ	
Sebeni ሰበኒ		Rehido ሬሂዶ	
Marua ማሩዋ		Debela ደበላ	
ida ኢዳ		Reita ሬይታ	

5. Apart from the sheep and goats that died, during the last one week, how many sheep and goats were sick? (give the number and disease) ባለፈው፡ አንድ፡ ሳምንት፡ ከሞቱት፡ በጎችና፡ ፍየሎች፡ ሌላ ምን ያህል፡ ፍየሎችና፡ በጎች፡ የታመሙ? ቁጥራቸውን ከበሽታው ይግለፁ.			
Lagaday ለጋዴይ		Moyu ሞዩ	
Anayee አናዬ		Bokole ቦኮሌ	
Gahsuru ጋሹሩ		Giregire ጊሬጊሬ	
Sebeni ሰበኒ		Rehido ሬሂዶ	
Marua ማሩዋ		Debela ደበላ	
ida ኢዳ		Reita ሬይታ	

6. During the last one week, how many sheep and goats did you give away? (give the number and the reason)

[illegible]

Lagaday [illegible]		Moyu [illegible]	
Anayee [illegible]		Bokole [illegible]	
Gahsuru [illegible]		Giregire [illegible]	
Sebeni [illegible]		Rehido [illegible]	
Marua [illegible]		Debela [illegible]	
ida [illegible]		Reita [illegible]	

7. During the last one week, how many sheep and goats did you receive as gifts?

[illegible]

Lagaday [illegible]		Moyu [illegible]	
Anayee [illegible]		Bokole [illegible]	
Gahsuru [illegible]		Giregire [illegible]	
Sebeni [illegible]		Rehido [illegible]	
Marua [illegible]		Debela [illegible]	
ida [illegible]		Reita [illegible]	

8. During the last one week, how many sheep and goats did you slaughter? (give number and reason)

[illegible]

Lagaday [illegible]		Moyu [illegible]	
Anayee [illegible]		Bokole [illegible]	
Gahsuru [illegible]		Giregire [illegible]	
Sebeni [illegible]		Rehido [illegible]	
Marua [illegible]		Debela [illegible]	
ida [illegible]		Reita [illegible]	

Remarks
