# Supplementary material for: Pastoralist knowledge of sheep and goat disease and implications for peste des petits ruminants virus control in the Afar Region of Ethiopia
Source: Prev Vet Med. 2020 Jan;174:104808. doi: 10.1016/j.prevetmed.2019.104808 (PMC6983938; doi:10.1016/j.prevetmed.2019.104808)
Supplement: Supplementary file 3 [file mmc3.docx]

Supplementary Information 3

Alphabetical list of Afar small ruminant disease terms recorded during the study

This list is provided as a quick reference with a brief description of the syndrome and a possible biomedical interpretation.

| Afar term | Literal translation | Brief description of syndrome | Possible biomedical syndrome or differential diagnoses |
| --- | --- | --- | --- |
| *Abale* | blood | blood in urine | haematuria |
| *Abeb* | NA | lameness, mouth lesions | foot-and-mouth disease |
| *Abitya* | NA | mandibular paralysis, hard stomach, constipation | Prosopis toxicity |
| *Afu delela* | mouth wound | lesions inside and around mouth | orf and other causes of mouth lesions |
| *Agara* | itching | skin disease | mange |
| *Aluley* | leech | leech | leech |
| *Andero* | fever | disease of cattle, sheep, goats – blood in urine, jaundice | haematuria and jaundice |
| *Aranwagit* | *aran* – sky, *wagit* – seeing  Looking to the sky | lacrimation, blindness, various neurological signs; looking to the sky, circling, paddling, vocalising. | Coenurosis, heartwater, other neurological diseases. |
| *Arbite* | bloated* | swollen stomach | bloat |
| *Armako* | NA | mandibular paralysis after eating Prosopis | Prosopis toxicity |
| *Asayta* | fever | fever, unwell, like malaria in people | systemic disease |
| *Asdaho* | red urine | red-coloured urine | haematuria |
| *Aysho-uruga* | grass diarrhoea | acute diarrhoea associated with wet, green grass | “nutritional” diarrhoea |
| *Baromeko* | NA | circling | neurological syndrome |
| *Bata’a* | down | animals do not eat or move and then die | more information required |
| *Biyakita* | disease | general term for disease | disease |
| *Bogo, bogo biyakita* | stomach, stomach disease | abdominal problem | abdominal disease |
| *Daariya* | NA | worms | gastro-intestinal worms |
| *Dalela* | wounds | skin lesions | skin lesions |
| *Dalohtay* | NA | death of weak/thin goats when rained on | death due to malnutrition/exposure |
| *Do’u* | lump | skin abscess or lump | abscess or other swelling |
| *Duduba* | swelling | abscess, lump, oedema or generalised swelling | abscess, oedema, inflammation |
| *Dugahabe* | the village is taken away | coughing, nasal discharge, mouth lesions, diarrhoea – disease of cattle, last seen about 10 years ago | rinderpest |
| *Fanache dalte* | Early birth | abortion | Abortion |
| *Fino* | NA | bloody diarrhoea, nasal discharge, dyspnoea | upper or lower respiratory tract infection, gastro-intestinal tract infection |
| *Firra* | NA | sudden death, bleeding from nose and other orifices | anthrax |
| *Furoda/fudoda* | NA | disease of eyes and lungs | Upper or lower respiratory tract infection, conjunctivitis, kerato-conjunctivitis |
| *Gesohabe* | the pen is taken away | lacrimation, nasal discharge, mouth lesions, diarrhoea | name given for PPR-like disease |
| *Gibiatu* | NA | large tick | tick |
| *Goson* | coughing | coughing | upper or lower respiratory tract infection |
| *Gublo* | lungs | lung disease | lower respiratory tract infection |
| *Hakuma* | scratching | scratching | pruritus |
| *Hamma* | NA | skin disease, wounds and cracks in skin of legs, occurring after rainy season | Dermatophilosis, mange, other skin disease |
| *Hanat biyakita* | milk disease | lamb/kid dies after drinking milk from sick mother | neonatal death |
| *Harufa* | NA | sores on sheep’s tail due to collection of faeces and urine in folds | urine/faecal scalding |
| *Harugi* | NA | death of new-born that drank colostrum and then died | neo-natal death |
| *Haysho biyakita* | urine disease | pain on urination, leaking urine | urolithiasis |
| *Hubya* | NA | very small tick | tick |
| *Iba, iba kosinta* | leg, limping | lameness | foot rot, infection or abscess in foot |
| *Iba’adu* | white legs | ticks | tick |
| *Inkata* | insects | lice | Lice |
| *Kaho, kahoenta* | coughing | coughing | upper or lower respiratory tract infection |
| *Karo* | unwell | sick, unwell | systemic disease |
| *Kilimi* | NA | ticks | tick |
| *Kirbi* | NA | disease associated with grazing in swampy areas – anaemia, diarrhoea, weight loss, | liver fluke |
| *Korboda* | stones on neck | external – skin nodules  internal – systemic disease | sheep and goat pox, other systemic disease |
| *Kos* | limp | lameness | foot rot, infection or abscess in foot |
| *Kutkuda* | NA | tick, mainly found on cattle | tick |
| *Lador, ladore* | cattle chooser (attacks cattle) | cattle disease – causes sudden death, swelling and bleeding from nose, mouth, anus | anthrax |
| *Mesengele* | lungs | lung disease | Lower respiratory tract infection |
| *Migda* | NA | sudden death, bleeding from nose and other orifices (used interchangeably with *firra*) | Anthrax |
| *Ndugulu* | sleepy | high mortality disease of cattle, with ocular and nasal discharge, diarrhoea | Rinderpest in cattle  Reported by one Animal Health Technician in the south of Afar to be a term for PPR disease small ruminants |
| *Roo* | NA | skin disease, similar to *korboda* | sheep and goat pox |
| *Sanak ofuwota* | lack of breath from the nose | noisy breathing due to blocked nose | blocked nostrils due to nasal discharge |
| *Sanaka* | *san* = nose | nasal discharge | nasal discharge |
| *Sandera* | NA | skin disease | Dermatophilosis |
| *Santiable* | nose blood | nasal discharge with blood, circling and stretching head back | nasal discharge, *Oestrus ovis* infection |
| *Silimi* | NA | ticks | ticks |
| *Slaytu biyak* | wind disease | various non-specific signs | More information required |
| *Solis* |  | unable to walk | More information required |
| *Sura gublo* | mucus lung | nasal discharge and lung disease | upper or lower respiratory tract infection |
| *Sura’ale* | mucus | nasal discharge | upper or lower respiratory tract infection |
| *Sura’atu* | mucus | nasal discharge | upper or lower respiratory tract infection |
| *Sura’atu gublo* | mucus lung | nasal discharge and lung disease | upper or lower respiratory tract infection |
| *Tafa* | NA | death of a weak animal struggling in the mud after it has rained | death due to malnutrition/exposure |
| *Ululu* | hunger* | malnutrition, starvation | death due to malnutrition |
| *Undahi* | slowly | diarrhoea syndrome | gastro-intestinal tract infection |
| *Undufeyta* | NA | lameness | foot rot, infection or abscess in foot |
| *Uruga* | diarrhoea | diarrhoea | gastro-intestinal tract infection |
| *Wahita* | shivering | shivering | shivering |
| *Waybo* | NA | external – skin nodules  internal – systemic disease | sheep and goat pox, other systemic disease |

*Parker, E. M. (2006) *English-Afar Dictionary*, USA: Afar Pastoralist Development Association, Dunwoody Press.
