# Supplementary material for: Pastoralist knowledge of sheep and goat disease and implications for peste des petits ruminants virus control in the Afar Region of Ethiopia
Source: Prev Vet Med. 2020 Jan;174:104808. doi: 10.1016/j.prevetmed.2019.104808 (PMC6983938; doi:10.1016/j.prevetmed.2019.104808)
Supplement: Supplementary file 4 [file mmc4.docx]

Supplementary information 4. Summary of commonly used Afar disease terms and biomedical interpretation

The common terms have been broadly categorised by body system affected.

| Category | Afar Disease term | Literal meaning | Main characteristics | Biomedical syndrome and/or differential diagnosis |
| --- | --- | --- | --- | --- |
| Respiratory | *Sura’atu, sura’ale* | Mucus | Nasal discharge (watery, mucoid or purulent), maybe associated with coughing, dyspnoea. | Upper or lower respiratory tract disease. Could be caused by bacteria, virus, parasites or mixed infection, including pasteurellosis, lungworm, CCPP and PPR, Nairobi sheep disease. |
|  | *Goson, kahoenta* | coughing | Coughing, usually with nasal discharge. Maybe associated with lacrimation, mouth lesions, dyspnoea or diarrhoea. |  |
|  | *Fododa, furoda* | NA | Lacrimation, conjunctivitis, nasal discharge, coughing, maybe with diarrhoea. Some cases have corneal opacity. Mainly affects younger animals, and sheep. |  |
|  | *Gublo, mesengele* | Lungs | Nasal discharge, coughing, dyspnoea, weakness. Maybe with lacrimation, fever, weight loss. Post mortem: lungs congested or collapsed, pleural effusion and adhesion. |  |
| Abdominal | *Uruga* | Diarrhoea | Various types of diarrhoea:   - Diarrhoea with blood, becomes thin and weak, - Chronic diarrhoea, - Acute, aysho-uruga (grass diarrhoea) – short fatal illness, occurring during rainy season, - Diarrhoea associated with browsing certain trees, - Mucoid diarrhoea, - Diarrhoea with worms.   Other signs include nasal discharge, coughing or lacrimation. | Gastro-intestinal infection due to acidosis, coccidiosis, entero-toxaemia, gastro-intestinal worms, PPR, Nairobi sheep disease. |
|  | *Undahi* | Slowly | Acute diarrhoea with abdominal swelling, or chronic diarrhoea with weight loss. There may be blood in the diarrhoea, and the diarrhoea may be black and foul-smelling. May be associated with sub-mandibular oedema, lacrimation or nasal discharge. | Gastro-intestinal infection  PPR |
|  | *Arbite* | Bloated | Swollen abdomen after eating bread or new grass after rain. | Bloat, intestinal blockage, acidosis. |
| Pox | *Korboda/ waybo* | Stones on neck | External type – nodules in the skin all over the body, and also may see fever, nasal discharge, conjunctivitis, lacrimation, mouth lesions, coughing, dyspnoea or weight loss.  Internal type – fever, nasal discharge, mouth lesions, coughing, abdominal pain, or diarrhoea. | Sheep and goat pox, other systemic disease |
| Skin disease | *Agara* | itching | Pruritic skin disease with crusting, skin-thickening and hair loss, affecting all parts of the body. Responds well to treatment. | Sarcoptic mange. |
|  | *Sandera* | NA | Wart-like exudative skin lesions and hair loss, affecting mainly the head, legs, genital area, but spreading to other parts of the body. Associated with tick bites. A chronic disease with no effective treatment. | Bacterial or parasitic skin disease, may be dermatophilosis or co-infections. |
|  | *Hamma* | NA | Scurfy pruritic skin with hair loss, affecting head and legs, occurring after rainy season. May become *sandera*. | Bacterial or parasitic skin disease, may be dermatophilosis or co-infections. |
|  | *Dalela* | Wounds | Wounds and other types of skin lesions | Various types of wounds or skin lesion. |
|  | *Afu-delay* | Mouth wounds | Wart-like lumps and sores inside and around the mouth of young animals. May also have nasal discharge, lacrimation, coughing or diarrhoea. | Orf and other causes of mouth lesions.  PPR |
|  | *Duduba* | Swelling | Abscess, lump, oedema or generalised swelling | Abscess, oedema, inflammation, caseous lymphadenitis |
|  | *Do’u* | Lump | Skin abscess or lump | Abscess or other localised swelling, caseous lymphadenitis |
| Ectoparasites | *Inkata* | Insects | Lice all over body, sucking blood and causing pruritus. | Lice |
|  | *Iba’adu* | White legs | Ticks, associated with lameness and skin disease. | Ticks |
|  | *Kilimi, silimi* | NA | Ticks, associated with lameness and skin disease | Ticks |
| Lameness | *Iba, iba kosinta* | Lameness | Interdigital lesions, swelling or abscess, or swelling/abscess above hoof. Associated with tick bites and thorns. May cause chronic lameness. | Foot rot, infection or abscess in foot |
|  | *Abeb* | NA | Disease of cattle, sheep and goats causing lameness in all four legs, mouth lesions and general sickness. | Foot-and-mouth disease |
| Reproductive | *Fanache dalte* | Early birth | Abortion, may be associated with other systemic diseases such as *korboda*. | Abortion, may be due to Brucellosis or another infection such as PPR or sheep and goat pox. |
| Urinary | *Abale* | Blood | Fever, blood in urine, jaundice. | Haematuria and jaundice, may be caused by Babesiosis or Anaplasmosis |
|  | *Andero* | Fever | Disease of cattle, sheep, goats causing blood in urine, jaundice | Haematuria and jaundice, may be caused by Babesiosis or Anaplasmosis |
| Neurological | *Aranwagit* | Looking to the sky | Lacrimation, blindness, various neurological signs; looking to the sky, circling, paddling, vocalising | Enterotoxaemia, heartwater, coenurosis, listeriosis, other neurological diseases. |
| Non-specific | *Slayti* | Wind | Generally unwell, with variety of other signs | Systemic disease |
|  | *Ululu* | Hunger | Malnutrition, starvation | Death due to malnutrition |
