# Supplementary material for: Pastoralist knowledge of sheep and goat disease and implications for peste des petits ruminants virus control in the Afar Region of Ethiopia
Source: Prev Vet Med. 2020 Jan;174:104808. doi: 10.1016/j.prevetmed.2019.104808 (PMC6983938; doi:10.1016/j.prevetmed.2019.104808)
Supplement: Supplementary file 5 [file mmc5.docx]

**Supplementary Information 5. Afar disease terms**

(extracted from Chapter 6 of Jones, B.A., 2018. Small ruminant production, marketing and health in the Afar Region of Ethioia: implications for control of infectious disease. PhD Thesis, Royal Veterinary College, University of London)

This document provides a description of terms used for small ruminant diseases in Chifra *woreda*, in the two study villages in Askoma and Halsaya *kebeles*, and other villages visited in Halsaya, Anderkelo, Geriro, Kusarale and Jarra *kebeles*. Most of the terms were related to clinical signs or affected body parts, so they have been organised by the main body system affected; respiratory, abdominal, skin disease, lameness, reproductive, urinary, neurological and non-specific. Observations on the use of these terms in two other *woredas* visited, Gewanne and Awash Fentale, and in other species, is also included. Terms that were recorded only once in Chifra, or were only recorded in other *woredas*, are described in the final section, together with additional terms recorded by previous studies.

Italicised alpha-numeric codes in parenthesis within the text indicate the specific interview or observation from which the data was obtained.

**1. General terms for disease**

A commonly used term for disease was *biyakita.* This was also used together with a body part to indicate disease of that part, such as *bogo biyakita;* *bogo* means stomach (*IH54*). *Karo* was a term used for a person or animal that was generally unwell (*IH34*), and was applied to cases that were sick but had no specific signs.

**2. Respiratory syndromes**

Commonly used terms for respiratory clinical signs were *sura’atu^^[[1]](#footnote-1)^^,* *sura’ale* and *sanak,* meaning nasal discharge (*IH3, IH30, ID2*), and *goson, kaho* or *kahoenta,* meaning coughing (*IH3, IH13*). Some of these terms were also the names of disease syndromes; *sura’atu, sura’ale,* and *goson. Gublo* and *mesengele* were Afar words for lungs and both were used to describe a disease syndrome affecting the lungs (*ID2, IH2-3, IH7, IH34, IJ1*). *Fododa* or *furoda* was a disease syndrome that affected the eyes and lungs (*ID10, ID18, IH62*). Terms were sometimes combined together to name a syndrome; *sura’atu-goson* (*IH3, IH38, IH43*), *sura’ale-goson* (*ID2*), *sura-gublo* (*IH2, IH12-13, IH21, IH35, IH50*), or *goson-gublo* (*IH5*).

*Sura’atu*, *goson, gublo* and *uruga* (diarrhoea) could occur together (*IH12-13, IH59, IH61*), especially in young animals (*IH35*). One respiratory syndrome could progress into another; *sura’atu* or *goson* could develop into *sura-gublo* (*IH7*); *gublo*, *goson* and *uruga* could progress from one to the other before death (*IH12*); *goson* could develop into *sura’atu* and then *mesengele* (IH27); or *mesengele* could become *goson* (*IH45*).

*Sura’atu, goson* and *gublo* were reported to occur during the drier and cooler weather in *gilal*, and during drought (Figure 1, *ID2, ID5, ID10, ID20, ID25, ID46, IH2, IH7, IH9, IH28, IH35, IH50, IH59, IH61*), which is supported by the results of the flock dynamics survey (Figure 2a). Most cases of *goson* occurred during mid-*gilal*, while *sura’atu* cases increased during *gilal* to peak in late *gilal*.

*Sura’atu*

The syndrome *sura’atu* was characterised by profuse nasal discharge;

*“It makes the animal release too much nasal discharge that swings in and around the nose.” (IH7),*

This could be accompanied by coughing or dyspnoea (*IH7, IH28*) and caused significant mortality (*IH2, IH9-10, IH12, IH14, IH18, IH20, IH30*). One person said that *sura’atu* affected the heart, while *gublo* affected the lungs (*IH34*). In the flock dynamics survey, *sura’atu* was the most frequent cause of sickness (35.1% of reported sick animals) and the second most frequent cause of death (27.3% reported deaths, Figure 4). Clinical cases were commonly seen in both villages (Figure 6) with signs of watery, mucoid or purulent nasal discharge, with or without coughing, lacrimation, dyspnoea and/or weight loss.

|  | *Sugum*  (small rains)  April | *Hagay*  (hot dry)  May-June | *Karma*  (main rains)  July-Aug | *Gilal*  (cool dry)  Sept-Mar |
| --- | --- | --- | --- | --- |
| rainfall | •••••  •••• |  | ••••••••••  •••••••• | ••• |
| *goson* | •• |  | ••••  •••• | ••••••••••  •••••••••• |
| *undahi* |  |  | •••  ••• | ••••••••••••  •••••••••••• |
| *sandera* |  |  | ••••••  •••••• | ••••••••••  •••••••• |
| *agara* |  |  | •••••  •••• | •••••••••••  •••••••••• |
| *inkata* | ••••  ••• |  |  | ••••••••••••  ••••••••••• |

Figure 1 Seasonal calendar of common sheep and goat diseases affecting Village B flocks

Prepared during a group interview (*ID11*). For each disease, 30 counters were distributed between the seasons to indicate relative incidence.

*Goson, Kahoenta*

*Goson* affected both sheep and goats and caused high mortality (*IH9, IH12-13, IH30*); one person said mortality was higher in goats (*IH12*). Animals affected with *sura’atu-goson*;

*“produce much nasal discharge through the nose and it makes them cough. The infected animal becomes so thin with less meat on the body. It produces a very small amount of milk. The eyes show great change. It coughs and has too much nasal discharge in the nose.” (IH3)*

*Goson* was said to be carried by the wind and passed from animal to animal (*IH34*).

In the flock dynamics survey, *goson,* or the less commonly used *kahoenta,* was the cause of 2.0% sick animals and 1.0% deaths. In both villages, clinical cases of *goson* or *sura-goson*/*sura’atu-goson* were the most frequently seen syndromes, affecting all ages of sheep and goats, with signs of coughing, usually with watery, mucoid or purulent nasal discharge, and sometimes lacrimation, mouth lesions, dyspnoea, or diarrhoea. Some cases died, especially young animals. Some cases had been sick for one month or more and were in poor body condition, and some cases of *goson* were associated with *korboda*/*waybo* (see below).

The terms *kaho* and *goson* were also used for a coughing syndrome of camels, which was ranked as an important camel disease (*ID1-2, ID43, IH41, IH65*), and in people *goson* was the term used for a cold (*IH34*). In Anderkelo, *goson* or *kahoenta* was also reported to be one of the main sheep and goat disease problems (*AK1*).

*Gublo, Mesengele*

*Gublo* or *mesengele* was a disease of the lungs that affected all ages of sheep, goats and cattle (*ID2, IH2-3, IH7, IH34, IJ1*);

*“Gublo has small flesh which if attacked becomes swollen and wounded. It releases nasal discharge through the nose and coughs. The whole gublo becomes invaded by wounds.” (ID2)*

*“First it attacks one lung of the animal and transfers to the second lung. Once the second lung is attacked and eaten by the disease, then the animal dies.” (IH5)*

The disease had a gradual onset with signs of nasal discharge, sometimes with blood, noisy breathing, feeling cold, and remaining standing but collapsing if forced to move (*ID2, IH45*). At slaughter the lungs were described as swollen and red or dark-coloured, with blood, water, pus and pleural adhesions (*ID32, IH28*).

*Gublo*, *sura-gublo* or *goson-gublo* caused high mortality in sheep and goats (*ID2, ID38, IH2-3, IH5, IH7, IH12-13, IH21, IH28, IH34-35, IH50*), and goats were affected more than sheep (*ID38, IH28*). Several flocks in Derekoma had been affected by *gublo* in the previous year (*ID2*);

*“He has lost many (to gublo). It is not that easy to speak about. All finished. Not only him, the people in the whole village have lost.”(ID2)*

Injectable oxytetracycline was commonly used to treat *gublo* (*ID25, GK1*), and if treatment was given early then animals could recover (*ID2*). *Gublo* was transmitted via wind or by contact between sick and healthy animals (*ID2, IH34*).

In the flock dynamics survey, 3.6% of sick animals had *gublo* and 2.8% of deaths were due to *gublo*. A few cases of *gublo* were seen in village B with signs of coughing, dyspnoea and tachypnoea, together with lacrimation, nasal discharge, with or without blood, and weight loss. Some had been sick for several months. In village A, there were a few cases of *mesengele* with lacrimation, nasal discharge, coughing and fever, but *sura-gublo* was more common, especially in the young flocks that had signs of nasal discharge, tachypnoea, dyspnoea, eye discharge and diarrhoea, sometimes with fever or mouth lesions (Figure 5).

a) respiratory syndromes

b) pox and abdominal syndromes

c) other syndromes

Figure 2 Flock dynamics survey; number of sheep and goat deaths and sickness by month in village A: Adjusted monthly totals, taking into account variation in the number of data collection days per month

a) Derekoma – respiratory syndromes b) Halsaya – respiratory syndromes

c) Derekoma – other common syndromes d) Halsaya – other common syndromes

Figure 3 Clinical cases examined; percentage of cases by disease term in each season

Figure 4 Flock dynamics survey; number of sheep and goat deaths and sickness by disease syndrome

Total number of sick and dead animals reported during Nov 2013 to Nov 2014 in 14 flocks in Village A


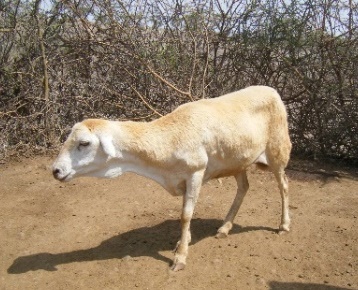

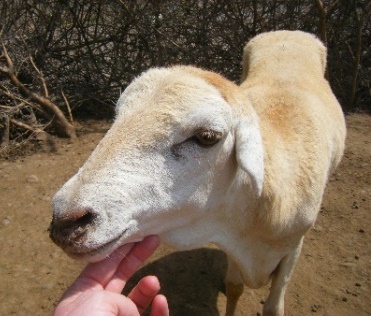

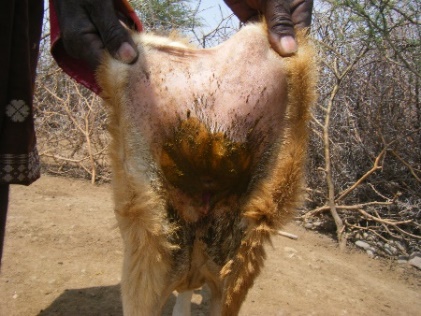


Figure 5 Adult sheep with *sura-gublo (IH7)*

a) dyspnoea – neck extended b) nasal discharge c) tail raised - diarrhoea soiling

*Fododa, Furoda*

*Fododa* or *furoda* was a syndrome with ocular signs, nasal discharge and coughing, sometimes with diarrhoea, especially in young sheep (*ID10, ID18, IH62*).

*“The disease attacks the eyes finally leading to blindness. It appears within the eye of the animal. It makes the goat lose the eye. The external part of the eye is fully covered and when you open the eye, you will see a wound. It also attacks the lung. After it attacks both sides of the lung, it attacks the eye and finally kills the goat. It mostly kills the young sheep. It kills many of them” (IH7)*

*Furoda* was differentiated from *sura’atu* and *gublo* as a disease of the eyes and lungs that mainly affected young animals (*IH35*). It occurred in those whose mothers had insufficient milk due to *ululu* (lack of food) (*IH46*), and spread among the young flock through contact (*ID18*).

In the flock dynamics survey *furoda* caused 0.6% of sick animals and 0.7% of deaths. Clinical cases were commonly seen in both villages during the study, mainly in groups of new-born and young sheep and goats, which showed a range of signs within each group including lacrimation, nasal discharge, coughing, diarrhoea, wart-like lumps around the mouth, mouth lesions and fever. In some cases the lacrimation was purulent and there was conjunctivitis or corneal opacity.

Similar descriptions of *fododa* were given in Jarra and Anderkelo (*IJ1, AK1*), but the term was not mentioned in other *woredas*.

**3. Abdominal syndromes**

The most common abdominal problem was diarrhoea. The Afar word for diarrhoea was *uruga* (*IH1*), which was used to describe the clinical sign of diarrhoea in diseases such as *undahi* (*IH1-3, IH5, IH7*), *sura-gublo* (*IH2*), or *waybo* (*ID76*), and was also used as the name of a disease syndrome for which diarrhoea was the main sign. The term *undahi,* meaning “slowly”, was the name of a diarrhoea syndrome (*IH2*), which some people said was the same as or like *uruga* (*IH3, ID2*), or was a type of *uruga* (*IH5, ID10*).

*Bogo biyakita* (sick stomach) was a more general term that was occasionally used for clinical cases with diarrhoea or abdominal discomfort (*ID32, ID38, ID82, IH54*).

Another abdominal problem was *arbite*, which means bloated, and was reported to be a problem during *karma* (*ID70-71*). Affected animals developed a swollen abdomen after eating new grass after rain, or from eating bread. No clinical cases of *arbite* were seen during the study, but one adult sheep was reported with *arbite* during the flock dynamics survey (*T13-52*).

a) Village B

b) Halsaya *kebele* including village A

Figure 6 Clinical cases examined during the field study

The diseases are colour coded according to the body system affected; respiratory – blue, abdominal – orange, pox – red, skin infection – green, wounds/abscesses – khaki, ectoparasites – black, lameness – purple, reproductive – yellow, non-specific – grey.

*Uruga*

The signs of *uruga* were diarrhoea, sometimes with blood, and a swollen stomach. The affected animal usually died (*IH2, IH7, IH11, IH15*). Several types of *uruga* were described. In *undahe-uruga* there was blood in the diarrhoea and the animal became thin and weak, and died after some time (*ID10, IH34*);

*“Blood comes out along with it (uruga) - some kind of fluid which looks like the liquid of a newly delivered animal, and blood comes out of the body of the animal” (IH12)*

Another type of *uruga* occurred in adult sheep and goats when browsing on certain trees. A chronic form of *uruga* lasted several months before death, and *aysho-uruga* (grass diarrhoea) was a short fatal illness associated with rain (*ID10, IH34, ID70, ID72a*);

*“the other type of uruga is one which appears during the time of rain season when the grass is wet and green. They call it grass uruga. It kills them instantly. Animals eat the grass, immediately their belly becomes filled with air, then it immediately dies.” (IH34)*

The diarrhoea could be pale, like nasal discharge (*IH13*), or there could be white material in the diarrhoea (*IH15, ID11*). One person described small (1cm long) *daariya* (worms) in the diarrhoea (*IH16*). There could also be signs of nasal discharge with blood, coughing with blood, and/or lacrimation (*IH2, IH7, IH12, IH16-17, IH30, IH43*), and the diseases *sura'atu, gublo* and *goson* were often associated with *uruga* (*IH13, IH18, IH30*).

Goats were more affected than sheep (*IH30, ID38*). Some people said all ages were affected (IH7), while others said it affected mainly adults (*IH2, IH17, IH30*). Kids and lambs could be affected when they drank the milk of an affected mother (*IH2, IH17*). There was said to be no treatment for *uruga* (*IH12*). Some animals recovered (*ID38*) but they could get it again at a later date (*IH4*3).

*Undahi*

*Undahi* was reported to be a common disease syndrome of sheep and goats (*IH1-3, IH18-20, ID2, ID5, ID19, ID20, ID25*), with signs of diarrhoea, abdominal swelling and death, or a more prolonged disease with weight loss and death after some time (*ID2, ID10, ID19, IH1-3, IH5, IH7*). Some people said the disease was more severe in sheep (*IH1-2, IH28, IH34*). Swelling of the hindquarters and throat was also described (*IH2, IH17*).

*“It attacks the stomach. The intestine too. It defecates diarrhoea and a small amount of blood too. It bleeds and feels cold. All of a sudden it dies.” (ID2)*

*“Most of the time our animals die because of uruga. They call the name of the disease undahi. It swells their belly and makes them release diarrhoea and die. This disease called undahi is one which finally turns in to uruga. It swells the tail of sheep, and this part of their body (indicating the throat) also swells” (IH2)*

*“It is one which makes animals release diarrhoea every now and then. Everywhere the infected animal goes, it releases diarrhoea. And slowly it dies.” (IH3)*

Some people described blood in the diarrhoea (*IH7, IH17, ID2, ID10, ID19*), one person said the diarrhoea was black (*IH28*), and another said it was like water with black bits in it (*IH34*), and had a bad smell (*IH28*);

*“No one can sit next to the undahi-infected animal. It stinks.” (IH34)*

*Undahi* could also have signs of lacrimation and/or nasal discharge (*ID19, IH17*).

Some people linked the name *undahi* (slowly) to the slow release of diarrhoea (*IH1*), and others to the slow death (*IH3*). It was frequently described as a chronic disease that made animals unproductive (*IH3, IH7, IH18-19, IH28, IH34, ID20*);

*“First, it weakens and makes the bones very thin. It also weakens the back of the animal. The sick animal becomes weaker and weaker. It does not produce milk or have meat. It also does not die quickly but remains weak for a long time.” (IH18)*

*“The animal becomes so thin. The meat of the body goes. The skin sticks on the body. No milk. There will be repetitive uruga and because of the uruga the milk dries. Uruga is the sign of undahi. The goat remains only with bones and skin. The sick goat discharges blood mixed with uruga. It attacks all sheep and goats. They all die finally. They cannot be used for any purpose.” (IH7)*

Both *uruga* and *undahi* were reported to occur during *karma* but were most common during the cooler weather of *gilal*, and during drought (Figure 1, *ID5, IH52, IH3, IH7, IH9*). At that time the animals were weak, thin and hungry, and *inkata* (ectoparasites) started to multiply (*IH3*). Cases of *undahi* occurred during *karma* because animals were weak after the dry season, then ate different types of grass that grew after rain, which caused watery brown diarrhoea, especially in adult goats, followed by rapid death or recovery (*ID57, ID64*).

In the flock dynamics survey, *uruga* was the most common cause of death (34.2%) and the second most common cause of sickness (31.4%), while *undahi* caused 7.4% of deaths and 7.7% sickness (Figure 4). *Uruga* was reported throughout the year, but increased in late *gilal* and *sugum*, while *undahi* occurred mainly from *sugum* to early *gilal* (Figure 2b).

Clinical cases of *uruga* were commonly seen during the study, especially during *karma* in village A (Figure 3d). In village B, *uruga* occurred more frequently in new-born and young sheep and goats, with signs of diarrhoea only (mucoid or pasty, greenish, or with blood), or with fever, lacrimation, nasal discharge, coughing or weight loss. In village A, it was more frequently seen in goats, especially groups of new-born and young animals. In adults the clinical signs were usually green or black watery diarrhoea with mucus and sometimes blood. In young animals there was usually a range of signs within the group, including black diarrhoea, fever, nasal discharge, lacrimation and coughing. A few animals had erosive or granulating mouth lesions on the gum, tongue or hard palate (Figures 7 and 8).

Only one case of *undahi* was examined; a young adult goat with *uruga-undahi* had been sick for one month with signs of greenish mucoid diarrhoea, lacrimation and nasal discharge, while the rest of the flock was not affected (*ID8*).

*Uruga* was described as a common disease problem in other Chifra *kebeles* (AK1, GK1), and in Awash Fentale (*AF14, AF5-7, AF9*). Similar descriptions of *undahi* were given in the other *kebeles* in Chifra (*IJ1, IK1, C3, IG1*), but this term was not reported in the other *woredas*.


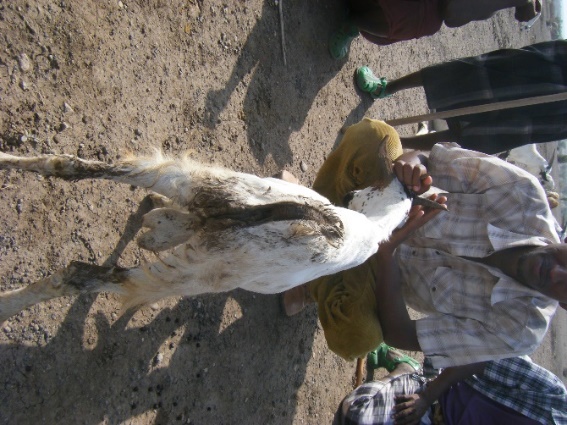

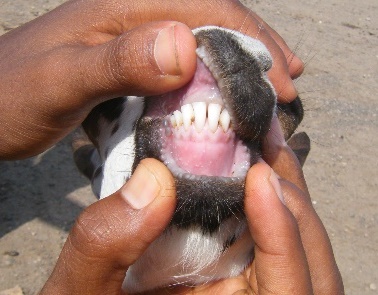


Figure 7 Young adult goat with watery diarrhoea and pin-point mouth lesions *(IH6)*


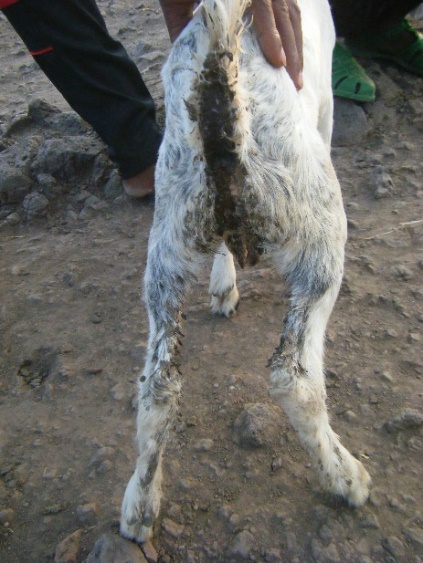

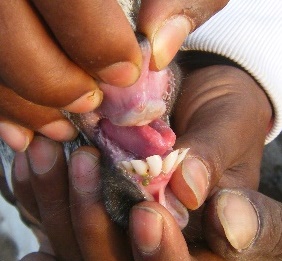


Figure 8 Young goat with *uruga*; diarrhoea, mouth lesions (sores on tongue, upper gum and hard palate), fever (*IH12*)

**4 Pox and related syndromes**

Two terms, *korboda* (or *kodboda*) and *waybo*, were used by almost all groups and households for a disease syndrome that was characterised by the typical skin lesions of sheep and goat pox. This was a common disease of sheep and goats and a major cause of death (*IH1-3, IH7, IH9, IH12, IH14-17, IH19, IH34, ID2, ID19, ID42*). The Afar word *kodbo* means “side of neck” and *daa* means “stone”, hence *kodboda* means “stones on side of neck”(*IH1*), describing the small stone-like lumps in the skin (*IH1-3*). People used the terms *korboda* and *waybo* interchangeably for the same disease (*IH1, IH7, IH9, IH19, IH25, ID2, ID72a, AK1*), but *korboda* was more commonly used in village A and *waybo* in village B.

Two types of *korboda* were described that occurred at the same time within a flock; external and internal (*IH27, IH31*). External *korboda* caused small swellings (*duduba*) in the skin all over the body (*IH16-17, IH20, IH28*) from which most animals recovered (*IH43, ID77, ID82*);

*“Kodboda is a disease that attacks all parts of the body of its victim. The wounds appear all over the body including on the eyes. The eye colour becomes completely changed and tears flow down and the wound appears inside the eye.” (IH15)*

The signs of internal *korboda* were *karo* (unwell) with variable signs such as fever, nasal discharge, mouth lesions, coughing, abdominal pain, or diarrhoea in young and adults (*IH26, ID19, ID76*).

*“It is a disease that attacks the body. For some, it attacks the external parts of their body and kills them. For some it attacks the internal part of their body. Internally it becomes like goson. It discharges diarrhoea.” (IH12)*

Animals with internal *korboda* were more likely to die than external cases (*IH27, IH34, IH43, ID76-77, ID82*). In some flocks more goats were affected than sheep (*ID77, ID81*), and in others, sheep were more affected than goats (*IH34, ID72a*). *Korboda* caused reduced milk production (*IH3, IH12, ID76*), and was associated with abortion (*IH12, ID19, ID77*) and death of new-born animals (*IH6, IH12, ID19*). One person in another village of Halsaya *kebele* estimated that one third of his goats and 40% of his sheep had been affected by *korboda,* of which half had died (16.7% of goats and 20% of sheep). The dams stopped producing milk so the young kids and lambs died (*IH6*). However, other people said that all their animals recovered (*IH30, ID81*). Animals that recovered were reported to be resistant to *korboda* in the future (*IH6, IH43*).

One person said *korboda* could spread through the environment (*IH30*), while another said that *korboda* occurred due to rain, wind and cold, for example when the flock was away from the home village and there was no *geso* to protect them (*ID72a*).


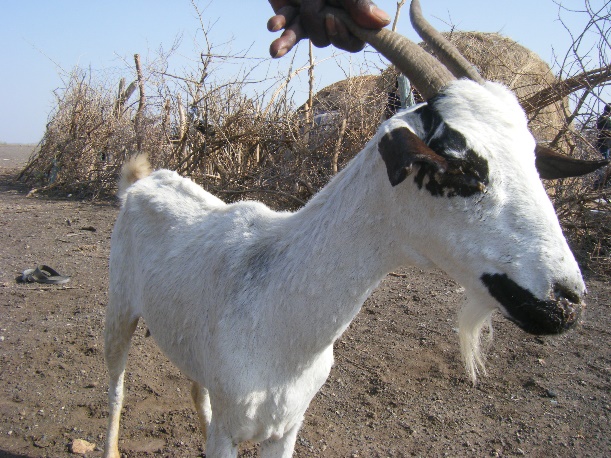

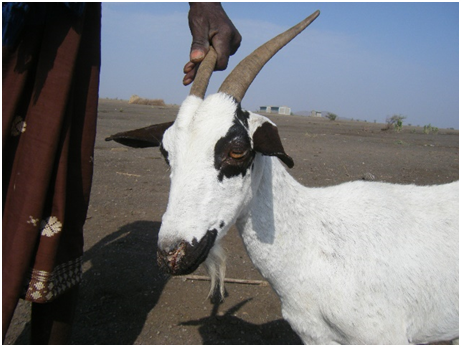


Figure 9 Adult goat with generalized pox lesions, ocular and nasal discharge with blood (*IH6*)


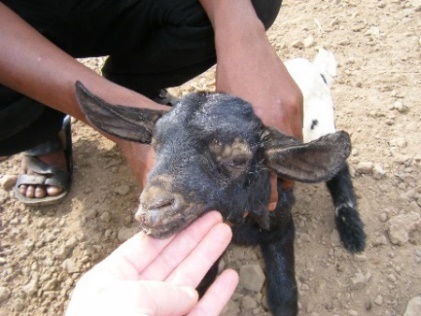

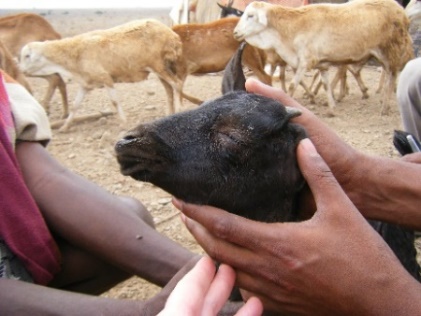

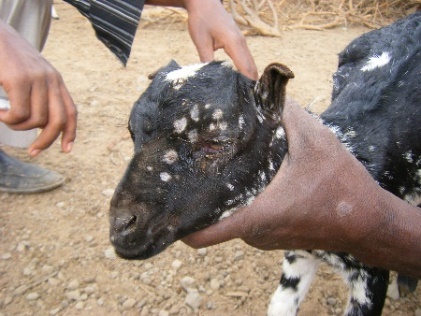


a) Early – pox around mouth, eyes, ears

b) Lacrimation and nasal discharge

c) Late – scabs all over face

Figure 10 Young goats affected by *korboda*, flock T11


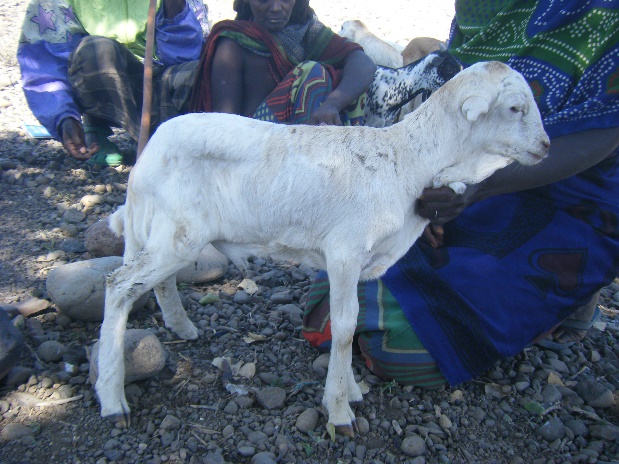

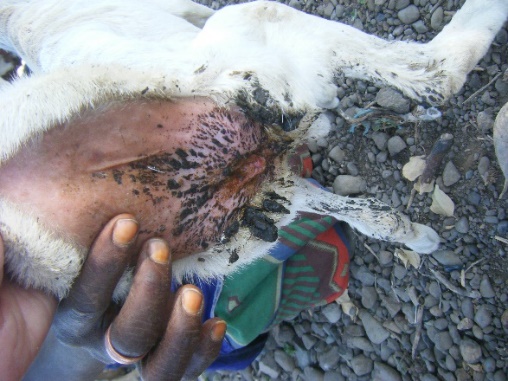


Figure 11 Young sheep with internal *korboda* - sick (*karo*) and diarrhoea (*IH27*)


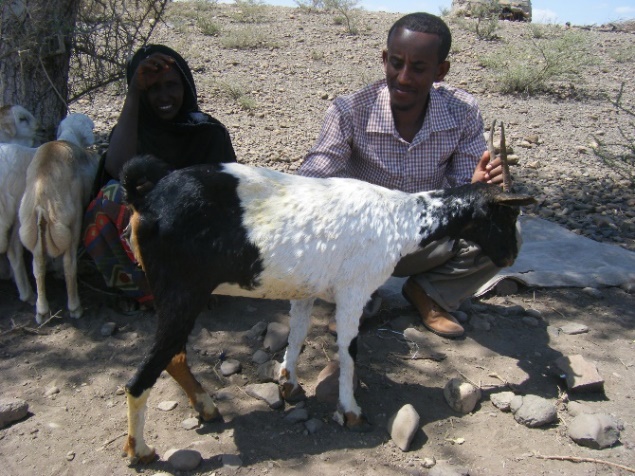

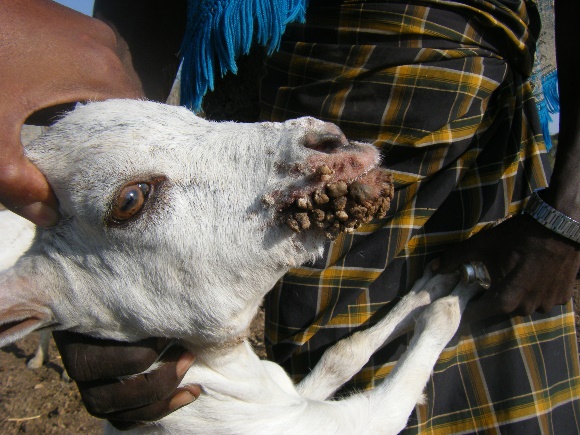


Figure 12 Adult goat with laboratory-confirmed sheep and goat pox

Figure 13 Young kid with *waybo* – nodules around the mouth

During the study period, cases of *korboda* were seen in October-November 2013 in village A and a neighbouring village, and the disease was reported to have been occurring since July (*IH30*). Cases decreased in late *gilal* (*IH39, IH43-44, IH47*), and no cases were seen during early or mid-*gilal* 2014 (*IH59, IH61*) (Figure 3d). This temporal pattern was seen in the flock dynamics survey (Figure 2b); 6.0% deaths and 7.8% of sickness were due to *korboda* (Figure 4).

In contrast, no cases of *korboda/waybo* were seen in village B until August 2014 when two flocks were affected (*ID50, ID57-59*), and many other flocks subsequently became infected (*ID63, ID72a, ID76-77, ID81-82, ID90*) (Figure 3c). The temporal difference in incidence between the villages may have been related to pox vaccination; many flocks in village B were vaccinated in May 2013, before the study, while village A flocks had received no vaccination for a number of years but were vaccinated during the study in March 2014.

The clinical cases of *korboda* examined were sheep and goats of all ages, with firm 1cm diameter cutaneous lumps all over the body, usually with nasal discharge, purulent eye discharge, and sometimes conjunctivitis and corneal opacity, mouth lesions, coughing, dyspnoea or weight loss (Figures 9-12). Signs were more severe in young animals, which were more likely to die. Early cases had fever, lacrimation and nasal discharge, and developed tufts of raised hair over the developing skin nodules. Later in the course of the disease, the lumps reduced, scabs appeared and then fell away to leave small patches of hair loss and healing skin. Some affected animals were reported to have aborted. The term *korboda* was also applied to cases with single lumps, or lumps that were localised to the face or head (Figure 13).

In flocks affected by external *korboda*, a few cases of internal *korboda* were examined that had fever together with diarrhoea and mouth lesions (Figure 11), or dyspnoea and nasal discharge.

One village A flock (T10) had cases they called *waybo*; an adult goat had typical signs of sheep and goat pox with skin nodules all over the body, fever (40.8°C) and nasal discharge (Figure 12), and a young adult sheep had swelling on one side of the face and granulating lesions on the lower gum. A nasal swab and scrape of a skin lesion was collected from the goat, and a scrape of gum lesion was taken from the sheep. These were put into virus transport media and submitted to NAHDIC (*IH25*). Both animals were capripox positive by real-time PCR.

*Korboda* was reported as a major disease in Anderkelo, Gewanne and Awash Fentale where they also described an external skin type from which animals usually recovered, and an internal type that usually led to death (*AK1, G3-5, AF5-8*). *Waybo* or *korboda* was also reported as a disease of cattle (*IH41*).

**5. Skin disease**

Several terms were used to describe disease syndromes causing skin lesions. *Agara*, which means “itching” (*IH2*), was a generalised pruritic condition, while *sandera* and *hamma* were more localised, with lesions usually occurring on the head, legs and genital areas (*IH7, IH15, ID16, ID23*).

*Dalela* means “wounds” (*IH2*). The term was applied to wounds caused by predator attack (*IH1*0), and when describing the signs of *sandera* and *agara*. The terms *dalela* and *afu-delay* (*afu* means “mouth”) were also applied to a syndrome of lesions around the mouths of young goats.

The term *duduba* means “swelling” and was used to describe skin abscesses and lumps (*ID38, ID74*), such as those associated with *korboda* (*IH28*), swelling associated with bite wounds (*ID11*), and sub-mandibular or generalised oedema (Figure 14). The term *do’u* was also used to describe abscesses or lumps in the skin (*ID5, ID18, ID38, ID57, ID84, IH43, IH61*) but was usually applied to more discrete lesions than *duduba*.


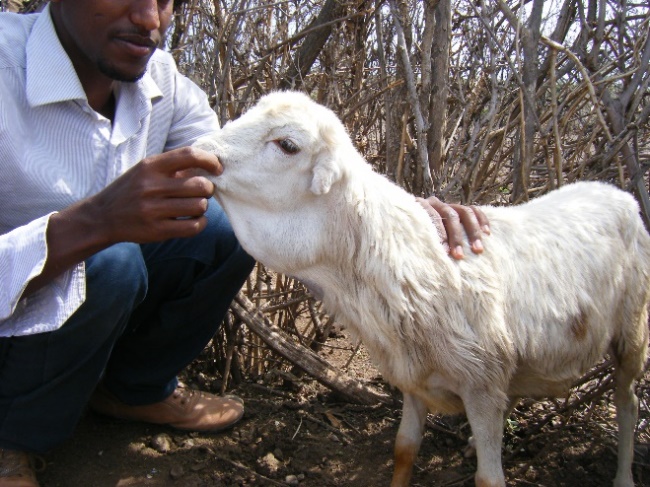


Figure 14 *Duduba* - sub-mandibular oedema (*ID47*)

*Sandera*

*Sandera* was frequently named as an important skin disease of sheep and goats during group and household interviews (*IH2-3, IH7, IH15, IH18, IH34, ID2-3, ID5, ID10, ID20*). It affected all ages (*IH3, ID2*), especially adults (*IH7*), and animals did not usually recover (*IH3, IH34, ID2*). There was reported to be no effective treatment (*ID2, ID10, ID20*), although in one case goat fat had been applied to affected skin (*ID12*). Cases of *sandera* were associated with *iba’adu* (ticks), with lesions appearing at the site of tick bites (*ID12, ID20*). It could also pass from one animal to another (*IH3*).

*Sandera* occurrence was linked to *agara*; if an animal developed *sandera* then later it would also get *agara* (*IH2*), or *vice versa* (*IH7, ID32*);

*“The first sign of sandera is that the animal gets affected by agara which is a skin disease. The animal remains with the agara. Sandera attacks the eyes, the legs, the ears of the animals and then it kills it. Once it is affected by sandera, it is finished. It is dead. It wounds all legs and the whole body of the animal.” (IH7)*

In village B in mid-*gilal* 2013, *sandera* was reported to be a current problem and was said to occur during *karma* and especially g*ilal* (*ID2-3*, Figure 1). Cases were quite commonly seen in mid and late *gilal* and *sugum* (Figure 3c)*,* and it was the second most common skin disease seen in village B after *afu-delay*. However, in the following year (*gilal* 2014), livestock keepers said there was no *sandera*, which they attributed to good rain in *karma* and early *gilal* producing good grass so the animals were healthy, compared to 2013 when there had been drought (*ID75, ID82*). Almost all cases were in adult goats, with signs of wart-like skin lesions with hair loss, exudation and scabs, which started on the lower leg, face and ears, perineum, teats or scrotum, and spread to other parts of the body; ventral abdomen and back (Figure 15-16). Most were chronic cases that had been sick for up to four months. Some cases had single lesions, and a few were young goats with orf-like lesions around the mouth (Figure 17).

In village A, *sandera* was said to be a new disease (*IH2*). A few cases were seen during the study, but in early *gilal* 2014 it was reported to have become a major problem because of the cold and rain (*IH59-61*). In the flock dynamics survey *sandera* was the most common skin disease, causing 0.7% deaths and 1.2% sickness, and mainly occurred in *sugum,* *hagay*, and early *gilal* 2014 (Figure 2c). It was the most commonly seen skin condition amongst the clinical cases in village A, with signs of granulomatous or verrucose lesions usually on the extremities; teat, prepuce, scrotum, perineum, vulva, face, ears or lower leg. Some had dorsal hair-loss with exudation, or ventral hair-loss and skin-thickening. *Sandera* was reported as a common skin problem in other Chifra *kebeles* (*IJ1, AK1, IK1, C3, GK1*), and was also said to be common in the neighbouring Amhara highland areas (*ID78*).


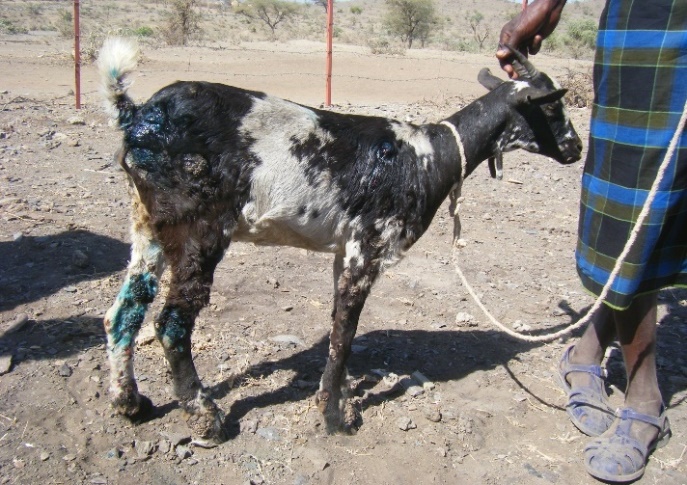

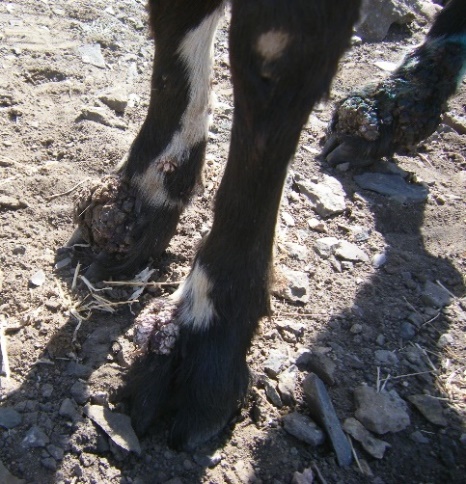


Figure 15 Adult goat with multiple lesions of *sandera*

Some of the lesions have been sprayed with oxytetracycline spray (*ID2*)


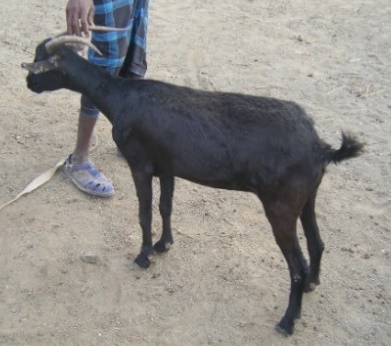

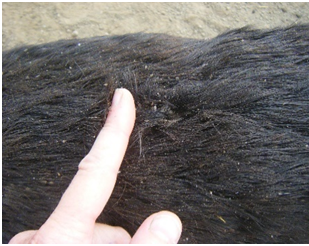

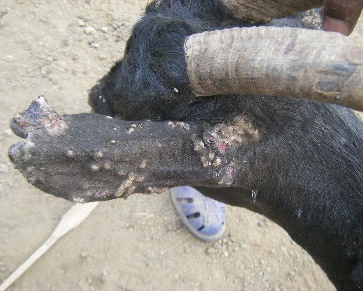


Figure 16 Adult goat with chronic *sandera* - crusts along back face and ears


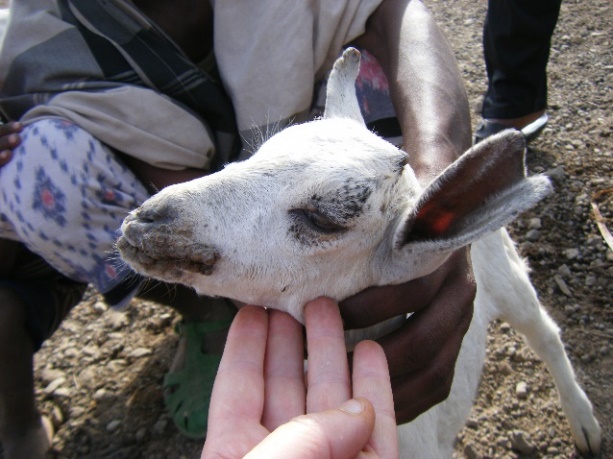


Figure 17 Young goat with orf-like lesions on lips, called *sandera* (T12, IH47)

*Hamma*

*Hamma* caused wounds and cracks in the skin of the legs (*ID1*6), and was reported to occur during *karma* after heavy rain that made the skin dry (*ID16, IH59*). *Hamma* could progress to *sandera* (*IH20, IH58*), but some people used the terms interchangeably (*ID16, ID23*). Cases were seen in both villages, with signs of scurfy itchy skin, broken hair and hair-loss that were progressing to become *sandera* or *agara*. The term was also applied to cases with crusty skin lesions on the muzzle, face and ears, and to a group of young goats with bleeding ulcerated lumps around the mouth and lacrimation. As for *sandera*, cases of *hamma* were reported to have increased in early-mid *gilal* 2014 due to the cool wet weather (*IH60-61*). Some people applied diesel to the lesions (*ID23*), or hair oil and salt (*ID38*).

*Agara*

*Agara* was a skin disease that caused pruritus leading to hair-loss and wounds all over the body, and could lead to death, but responded well to treatment (*IH2-3, IH7, IH15, IH34, ID17*). It affected goats more than sheep (*ID2, IH34*).

*“Agara removes the hair of the animal and it develops cracks gradually. However, once the crack is seen, if you put some medicine on it, it heals quickly. It does not spare any part of the body including the eyes.” (ID2)*

As for the other skin diseases, *agara* occurred in *karma* but was most common in *gilal* (*ID20, ID71,* Figure 1), and was caused by *iba’adu* (*ID20*). As mentioned above, *agara* was linked with *sandera,* and *hamma* could progress to *agara* (*IH58*). The livestock keepers purchased treatment, *agarale*, which was poured over the affected animal (*IH3, ID2*). They also used oxytetracycline, ivomectin and applied diesel (*ID17, ID36*).


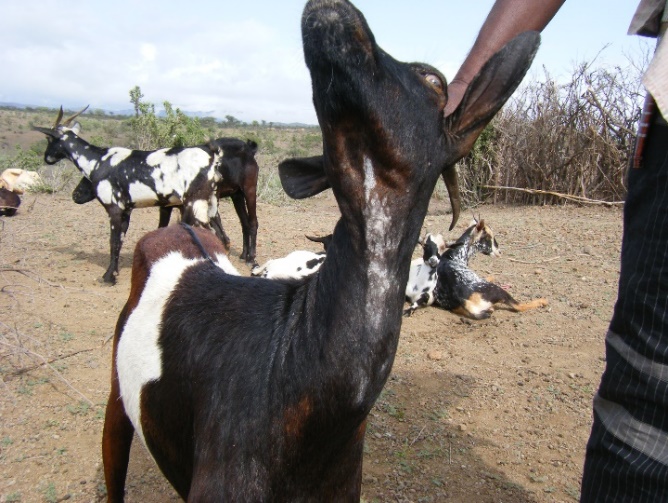


Figure 18 *Agara* in an adult goat - hair-loss and skin-thickening on ventral neck

In the flock dynamics survey, *agara* was reported as a cause of sickness only in goats and did not cause any deaths. Clinical cases were seen in both villages, with signs of itching, patches of broken hair, hair-loss, rough flaky skin, and exuding lesions with crusts. The signs were generalised or localised around the mouth, nose, ventral neck or scrotum (Figure 18). A few animals had orf-like lumps around the mouth and crusty lesions on the body. The term was also applied to an animal with a scurfy coat and louse eggs on the hair, and to animals that were recovering from *korboda*.

The other Chifra *kebeles* also listed *agara* as an important skin disease (*IJ1, IK1, C3, AK1*), and it was reported in Awash Fentale (*AF3, AF6-7*). *Agara* was also named as a disease of camels (*ID2, IH7, ID36*).

*Dalela, Afu delay*

*Dalela* (wounds) was a general term that was used for skin lesions and wounds, but *dalela* or *afu-delay* (mouth wounds) was also applied to a syndrome of orf-like mouth lesions in new-born and young goats, which varied from granulomatous lesions on the lips and gums (*ID40*), to large nodules on the muzzle and lips and gum ulceration (*IH26,* Figure 19) and foul-smelling wounds (*IH60*). Some also had nasal discharge, lacrimation, coughing or diarrhoea, and some died (*ID44, ID46*). One owner attributed the lesions to thorns (ID44).

In village B, cases were commonly seen in young flocks, especially in *sugum* and *karma*, and it was the most commonly seen skin disease (Figure 3c). However, in village A there were no reports of sickness or death due to *afu-delay* or *dalela* in the flock dynamics survey, and the terms were only applied to a few clinical cases in other villages in Halsaya.


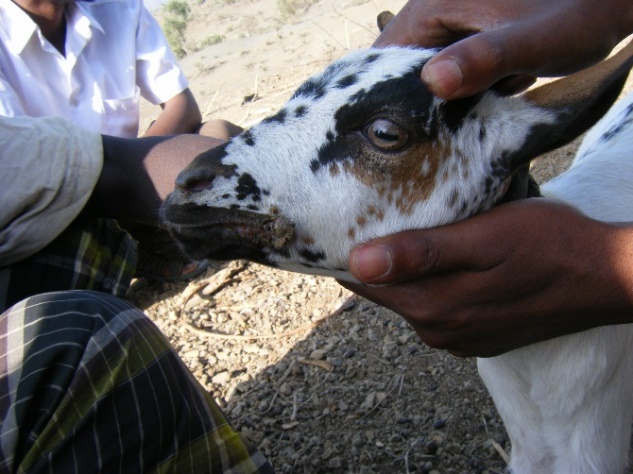

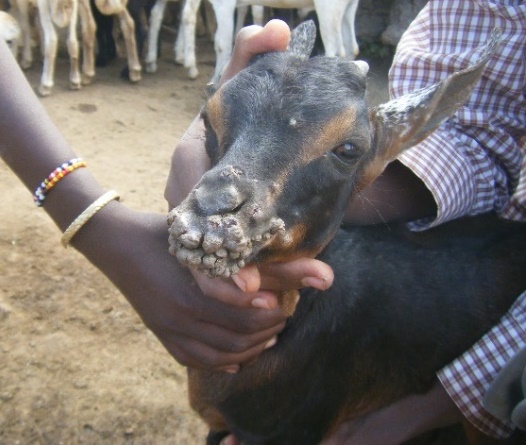


a) lesions on lips and mouth (*ID40*) b) large nodules around muzzle (*IH26*)

Figure 19 Young goats with *dalela*

**6. Ectoparasites**

There were several terms for ectoparasites; *inkata* means “insects” and was used for small “insects” that lived in the hair, while *iba’adu*, *kilimi* and *silimi* were terms for different types of ticks.

*Inkata*

*Inkata* were described by the livestock keepers as small insects that multiplied all over the body and sucked blood, causing scratching (*IH2-3, IH5, IH7, IH25*). Humans could also have *inkata* in their hair (*IH25*).

*“The production of inkata takes place inside the hair of the animal. Through time, the inkata make the animal very skinny. It neither bleeds nor does it produce milk.” (IH3)*

The market value of affected animals was reduced because they were thin and anaemic (*IH25*). *Inkata* were associated with late *gilal* and drought (*IH3, IH7, IH9, IH25*, Figure 1). Infestation with *inkata* predisposed animals to other disease problems;

*“inkata outbreaks happen whenever drought happens and consequently, diseases like sura’atu, goson, gublo, undahi, and others attack the animal. The inkata sucks all the blood of the animal and finally kills it.” (IH9)*

The livestock keepers used *sumi* (chemical) to treat *inkata*, such as washing the animals with diluted diazinon (*IH3, IH25, IH39*). In February-March 2014, all livestock keepers in both villages were complaining of *inkata* and requesting *sumi* to treat their flocks (*ID32, ID38, ID42, IH47*). There were also a few reports of *inkata* in August (*ID55*) and November 2014 (*ID77*). In the flock dynamics survey there were very few reports of death (0.1%) or sickness (0.1%) due to *inkata*, but clinical cases were seen in village B in late *gilal* when whole flocks, especially young animals, had louse eggs on the hair, hair-loss*,* and general sickness and diarrhoea (Figure 3c). Cases were seen in village A in *karma* and early *gilal* 2014, with eggs on the hair, itching, and rough hair (Figure 3d).

Calves were also affected by *inkata* (*ID43*). *Inkata* were reported to be a common problem in the dry season in Anderkelo (*AK1*), but were not mentioned in other Chifra *kebeles*, or in the other *woredas*.

*Iba’adu, Kilimi, Silimi*

*Iba’adu*, *kilimi* and *silimi* were terms for ticks. *Iba’adu^^[[2]](#footnote-2)^^* were a problem of sheep and goats that could cause death (*IH9, IH18*), and occurred throughout the year, especially during drought (*ID20, IH52*). *Iba’adu* were reported to cause *sandera* and *agara*, with lesions developing at the site of tick bites (*ID20*). *Kilimi* were smaller than *iba’adu* (*IH8*), and were reported to be a major problem in late *gilal* (*IH47*), and in *karma* (*ID55*). *Kilimi* were thought to be the underlying cause of lameness that occurred after rain and caused major losses (*ID56*). *Kilimi* also affected camels and cattle (*ID42-43*). *Silimi* were also smaller than *iba’adu* (*ID3*), and like *kilimi* were reported to be a major problem in late *gilal* (*ID19, ID42*).

In the flock dynamics survey, *iba’adu* was reported as a cause of 0.4% sickness and 0.1% deaths, while *kilimi* and *silimi* were the cause of 0.1% and 0.2% of deaths respectively (Figure 3). *Iba’adu* were frequently seen when examining clinical cases, especially lameness when ticks were found in the interdigital space, or on the scrotum or other sites associated with *sandera*. Some clinical cases of *karo* were attributed to *kilimi*, as well as cases of lameness, mastitis and *agara* on the lower leg.

*Kilimi* were mentioned in other Chifra *kebeles* as a problem in *gilal,* and as a cause of *sandera* and *undefeyta* (lameness, *AK1, IJ1, C3*).

**7. Lameness**

*Iba, iba kosinta*

*Iba* means “leg” and *kos* means “limp” (*IH3*). *Iba kosinta* means lameness but people frequently used *iba* to indicate that an animal was lame (*ID2*). *Iba* was a common problem of sheep and goats (*IH2-3, IH13, IH18, ID2, ID55*).

*“The disease eats the middle section of their foot. It makes their hooves grow long and makes them walk like that. Finally, the foot swells and it becomes so big.” (IH3)*

*Iba* affected one or more feet, causing swelling and lesions above the hoof and in the inter-digital space. It could spread to cause *gublo* or generalised sickness (*IH3, ID56*), and made animals vulnerable to predators (*IH15*). There was an increased incidence of *iba* during the rainy season, which livestock keepers thought was caused by *kilimi* (*IH52, IH56, ID60-61, ID63-64, ID72*), and there continued to be cases during *gilal* (*IH59, ID76, ID80*). It was treated by killing *inkata* and *iba’adu* with insecticides (*IH3*) or kerosene (*IH35*).

In the flock dynamics survey, *iba* caused 3.5% of deaths and 6.3% of sickness (Figure 4), and showed a slight increase in incidence in *sugum* and *hagay* and a much greater increase in *karma* and early-mid *gilal*. Clinical cases were commonly seen in both villages, especially in *karma* and early-mid *gilal* (Figure 3c and d), and in village A it was one of the most common clinical syndromes seen (Figure 6). Cases had interdigital lesions; a wound, sore, granuloma or abscess, or hot painful swelling above the coronet that developed into an abscess. Some lesions had maggots. The cases were associated with tick bites, thorns, previous sickness due to *waybo* or *gublo*, and in one case, a burn. Some cases had been chronically lame for up to 6 months with muscle wasting and overgrowth of the hoof.

*Iba* was also a problem in Geriro *kebele* in *karma* (*IG1*). In Awash Fentale, they used the term *kos* for lameness (*AF6, AF7*).

*Abeb*

In village A, *abeb* was described as an important disease of cattle, sheep and goats (*IH2-3, IH12, IH34, IH41*), but it was not mentioned in village B. Affected animals suddenly became lame in all four legs, were “sick in the stomach”, and developed wounds in the mouth, which prevented eating (*IH12, IH34*). There was no effective treatment for *abeb*, but they applied honey to the wounds (*IH41*). No clinical cases of *abeb* were seen during the study and none were reported during the flock survey. *Abeb* was also mentioned in Anderkelo, Gewanne and Awash Fentale (*AK1, G3-5, AF5-6*).

**8 Reproductive problems**

*Fanache dalte* means “early birth”. The term was used for animals that had aborted, and it was mentioned as a clinical sign of some diseases. Livestock owners said that their flocks usually experienced one or two abortions per year (*ID38, IH32*).

In village B, during September to November 2013, 17 goats aborted in four flocks (*ID11, ID15, ID17, ID19, ID22-24*). In one flock, six out of 36 breeding goats aborted (16.7%), and the other three flocks had two to three abortions each. One owner said that 20 out of his 30 goats had aborted during the previous *sugum* (April 2013). Blood samples from five recently-aborted animals from three flocks were submitted to the Regional Veterinary Laboratory, and all were strongly positive for brucellosis antibody by the rose Bengal test^^[[3]](#footnote-3)^^. Five abortions were reported in three other flocks in February-March 2014 (*ID32, ID38, ID43*), and the outbreak of *waybo* in August-November 2014 was reported to have caused many abortions (*ID73, ID77, ID*87).

In village A, sporadic abortions were seen in several flocks during the course of the study (*IH20, IH40, IH42, IH58, IH60*). However, in November 2013, seven goats in flock T4 aborted within four days and an adult male goat had a very swollen testicle (*IH32*). A nearby flock (T14) also reported ten abortions in the previous week (*IH37*).

From the flock dynamics survey results, the estimated annual abortion rate per breeding female was 4.6% in goats and 3.3% in sheep. Abortions were reported by 10 out of the 14 flocks, a median of three abortions per flock (range 0-12). However, this was likely to be an underestimate because some abortions may be undetected. Three flocks reported deaths of dams due to abortion, and four flocks had sick animals due to abortion.

In village B, 20 goats that had aborted one day to several weeks earlier were examined, and in village A two sheep and five goats were examined. They ranged in parity from first to fourth. Some had shown no signs of illness prior to aborting but some were *karo* (unwell). Some of the aborted foetuses had hair and some did not (Figure 20). Some of the animals had vaginal discharge, decreased appetite, diarrhoea, nasal discharge or *karo*. One had a retained placenta.


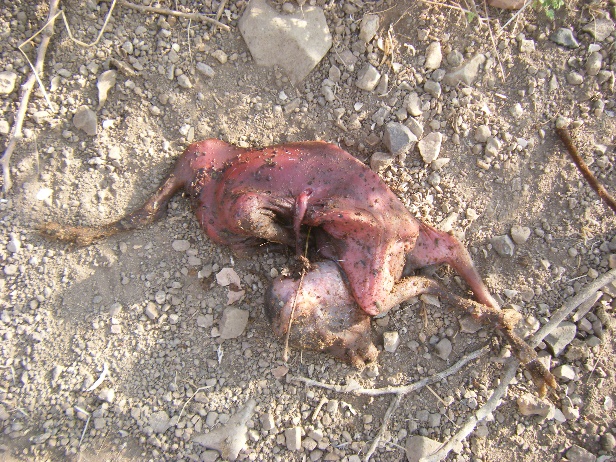


Figure 20 Aborted goat foetus (*ID17*)

**9 Urinary problems**

*Abale*

*Abale* means “blood”. *Abale* was reported as a small ruminant disease problem during interviews in Adara (*IH3*), Atkafala (*IH7*) and Lahilala (*IH34*), but not in the two study villages. No cases were seen during the study period and none were reported during the flock dynamics survey. *Abale* was reported to occur during the rainy season, and was associated with drinking rainwater or grazing wet grass (*IH3, IH7*). It was more common in goats than sheep, and mortality was high (*IH3, IH7*). The main signs were fever, blood in the urine, and yellow-green eyes and meat (*IH3, IH7, IH34*). A similar description was given in other Chifra *kebeles* (*IJ1, C3*), and in one *kebele* in Awash Fentale (*AF8*).

*Andero*

*Andero* means “fever” (*IH34*), and was a term used for malaria in humans (*G4*). It was mentioned as a small ruminant disease during the village B group interview (*ID2*), but no cases were seen during the study and none were reported in the flock dynamics survey. During an interview in one village in Halsaya, *andero* was described as a disease of cattle, causing blood in the urine and yellow-green eyes, which could be transmitted to people via milk (*IH34*). One livestock keeper lost seven cattle due to *andero* while they were grazing at the Mille River, with signs of lethargy, recumbency and death (*IH53*). Similar descriptions of *andero* in sheep, goats and cattle were given in Kusarale (*IK1*), Gewanne (*G4*), and Awash Fentale (*AF6*).

**10. Neurological syndrome**

*Aranwagit*

*Aran* means “sky” and *wagit* means “seeing”; *aranwagit* therefore translates as “looking to the sky”. This term was only mentioned during the village A group interview (*IH2*) and by one person in a neighbouring village (*IH62*), and no clinical cases were seen during the study period. It was also described in other Chifra *kebeles* (*IJ1, AK1*). The main signs seemed to be neurological; blindness, star-gazing, circling, paddling and vocalising, together with lacrimation. It was said to occur sporadically in individual animals, attacking the eyes, so that the animal looked to the sky and ran away (*IH2*). In Lahilala, a young flock was reported to have been affected during *sugum* with signs of looking to the sky, paddling, swollen ears and eyes, and blindness. Three out of seven affected animals died (*IH62*).

**11. Non-specific terms**

*Slayti, Slaytu biyak*

*Slayti* means “wind”; *slaytu biyak* means “wind disease”. One person said *slayti* described a sick animal with no obvious signs (*IH12*), but another person said it described an animal that fell over and paddled its legs (*IH65*). A thin, anaemic sheep with nasal discharge and skin lesions was described as having *slaytu biyak* that was becoming *undahi* (*IH12*). A young lamb that was close to death with no specific signs was sick due to *slayti* (*IH53*). *Slayti* was used for a case where an adult goat had fluid swellings on the anterior carpi (*ID53*). In the flock dynamics survey, *slayti* caused 0.1% deaths and 0.05% of sickness.

*Ululu*

*Ululu* is the term for famine or starvation, and can also mean lack of forage and drought. It was named as one of the common problems affecting flocks in late *gilal* (*ID42, ID46*). *Ululu* was explained as a young animal becoming thin and dying because its mother was not getting enough food and was not producing enough milk (*IH43*). In the flock dynamics survey it caused 5.9% deaths and 1.0% sickness, mostly in new-born sheep and goats in February and March (late *gilal*).

Some additional disease terms were recorded during the flock dynamics survey that were not reported during interviews or clinical examinations. The term, *dalotay*, was a cause of death during *karma* (0.9% deaths). It was described as the death of thin, weak animals after they had been rained on (*T1-24, T8-25, T9-25*). *Hanat biyakita* was the death of a new-born after drinking milk from its sick mother, *harugi* was the death of a new-born after drinking colostrum, *robo* was death due to rain, *sumi* was sickness after applying *sumi*, and *tafa* was the death of a weak animal due to struggling in the mud after it had rained in the night.

**12. Terms only used once in Chifra**

*Aluley:* is the word for leech. During flock examination, the owner found a sheep with a leech attached to her tongue and immediately removed it. He said they get *aluley* when drinking water (*ID18*).

*Baromeko: a* disease called *baromeko* was described in Anderkelo that they said was similar to *aranwagit*, causing circling (*AK1*).

*Bata’a:* means “down” and was used by one person to describe a syndrome that he said was the same as *undahi* in which animals did not move or eat, and then died. It affected many sheep and goats in Halsaya during *sugum* and *karma* (*IH52*).

*Hanat biyakita:* A lamb or kid dies after drinking milk from its mother who is sick (*T5-57*).

*Harufa: w*ounds on tail due to collection of faeces and urine (*ID83*).

*Harugi:* used to describe the death of a new-born lamb or kid that drank colostrum and then died (*IH65*).

*Haysho biyakita: haysho* means urine, hence *haysho biyakita* means “urine disease. One affected goat was seen that had pain on urination and leakage of urine from the penis (*ID18*).

*Sanak ofuwota: sanak ofuwota*, literally meaning “lack of breath from the nose”, was used to describe a blocked nose causing noisy breathing. (*ID31*).

*Tafa:* the name given to the death of a weak animal struggling in the mud after it has rained (*IH52*).

*Undefeyta: undefeyta* was another term that was used for a foot problem causing lameness (*IH7, IH14*). In Chifra kebele, one case was seen in an emaciated recumbent adult sheep with a very swollen lower hind leg (*C3*). They said that tick bites had caused wounds that then swelled. Gari *et al* (2015) described *undefeta* as lameness that affected many animals in wet conditions, causing severe interdigital lesions.

*Wahita:* means shivering and was applied to a sick adult sheep (*ID85*).

**13. Terms used in other *woredas***

Below are some other disease terms that were not heard in Chifra but only in other parts of Afar.

*Abitya: a*fter feeding on Prosopis, the animal stops defecating and has a hard stomach. It develops mandibular paralysis (*armako)* (*G4*).

*Armako: m*andibular paralysis that develops after feeding on Prosopis (*G4*).

*Asdaho: asdaho* was a term used in Awash Fentale, which means “red urine” (*AF5-7*). It was reported to be the same as *andero* (*AF6*). The term was not used in Chifra *woreda*, it is therefore possible that this is a synonym for *abale*. Gari *et al* (2015), indicate that *asdaho* and *abali* are the same disease syndrome.

*Fino:* in Gewanne market, they described a disease called *fino* that caused bloody diarrhoea and death in goats of all ages including suckling kids. When prompted, there were also mouth and nose discharge, lacrimation, shouting, difficulty breathing, occurring in all three kebeles at the time (*G5*).

*Firra, Migda: firra* was a term used only in Awash Fentale for a disease that affected cattle and other species including humans, and caused sudden death with bleeding from the nose and other orifices (*AF2,* *AF5, AF7*). There had been an outbreak the previous year in several *woredas*. *Migda* was a term for the same disease (*AF7*). Gari *et al* (2015) described *migda* as a disease of sheep with swelling on the shoulder and rough hair coat, while *firah* was a disease of sheep causing bleeding from the mouth and nose.

*Kirbi: kirbi* was used for a disease syndrome in Gewanne and Awash Fentale (*G4, AF2*), that was associated with grazing in swampy areas (*G4, AF2*). The term was used in one village of Halsaya to describe a disease of cattle that is associated with areas like Afambo where there are flooded areas near the river where cattle graze (*IH34*). Mariner et al (1994) reported that *kirbi* was liver fluke, associated with irrigated or flooded areas. Dagnatchew (2001) reported that *kirbi* was disease found in south Afar with signs of pale mucous membranes, diarrhoea, sub-mandibular oedema, weight loss, and small flat worms in the gall bladder. Gari *et al* (2015) give a similar description.

*Ladore: a* disease of cattle disease that causes sudden death, swelling of the neck, and bleeding from nose, mouth and anus (*ID2, IH34*). The term means “cattle chooser”. Mariner et al (1994) reported that it kills the best animal in a herd, and also affects people and donkeys. The signs were sudden death of healthy animals, bloated carcass and bloody discharge from the nostrils, anus, and mouth. The blood does not clot. It was reported to be anthrax.

*Ndugulu: ndugulu* was a term heard in Gewanne that was used for a disease of young cattle with high mortality causing lacrimation, drooping head and diarrhoea (*G3*). In Awash Fentale, one group said that *ndugulu* was a disease of cattle affecting the eyes and mouth, causing diarrhoea and death, which had not been seen for ten years (*AF7*). This was interpreted to be rinderpest. An AHA in Awash Fentale said that *ndugulu* was the local name for PPR and that the word meant “sleepy”, describing droopy ears and sleepy eyes (*AF3*). During one group interview, the term was heard when they described “internal” *korboda* as being like *ndugulu* with lacrimation, nasal and oral discharge, bloody diarrhoea and abortion (*AF5*). In another interview, when asked specifically about diseases with signs of lacrimation, nasal discharge, mouth lesions and diarrhoea they named *ndugulu* as a disease occurring in sheep but especially goats that affected the eyes and caused diarrhoea before death (*AF6*).

*Roo: roo* was named as a skin disease of sheep, goats and cattle that was similar to *korboda* (*AF6*)

*Santiable: santiable* (literally “nose blood”) was reported as a new disease with signs of nasal discharge with blood, circling and stretching the head back (*AF6*).

*Solis: solis* was a term used in Awash Fentale for a disease or sheep where they were unable to walk (*AF5, AF8*).

*Sole:* was a term used to describe emaciation, gradual weakness and death in sheep (Gari *et al* 2015).

**14. Comparison with Afar disease terms described by other authors**

**Respiratory syndromes**

In this study, *sura’atu* was a very common term, and *sura* and *sura’ale* were also used. Mariner *et al.* (1994) used a similar term, *surahi,* for a syndrome of coughing and nasal discharge that occurred more frequently in the dry season. Dagnatchew (2001) described *sura’ali* as a contagious disease with signs of coughing, nasal discharge, emaciation and death, with marbled lungs, and pleural fluid and adhesions. Gari *et al.* (2015) used the term *surota* for a pneumonia syndrome of goats with signs of coughing and nasal discharge that occurred during cold wet weather, while for sheep they reported that *surota* and g*oson* were synonyms for *foroda,* a transmissible broncho-pneumonia syndrome with signs of fever, coughing, blocked nose, and emaciation, which occurred during drought. Mariner *et al.* (1994) described *fududa* or *gosoni* as a pneumonia syndrome of lambs and kids with high morbidity and mortality and signs of coughing, inflamed lungs, difficulty walking and loss of condition. Dagnatchew (2001) described *feduda* and *endahi* as terms for the same syndrome; a contagious disease with signs of nasal discharge, coughing, dyspnoea, recumbency and death, with hepatisation and marbling of the lungs, and pleural adhesions. None of these studies mention the ocular signs of *furoda* that were commonly reported and observed during this study, and this study found that *furoda* had different characteristics from *undahi*, but had some similarities with *sura’atu* and *goson*. Mariner *et al.* (1994) and Gari *et al.* (2015) gave similar descriptions of *gublo* as in this study, but said it was a disease of goats and suggested it was CCPP. Gari *et al.* (2015) also reported *mesengele* to be a synonym of *gublo.*

**Abdominal syndromes**

As in this study, Mariner et al. (1994) described several types of uruga, including bloody diarrhoea and green watery diarrhoea, the latter occurring during the rainy season, causing death within a week, or chronic weight loss and slow recovery. Dagnatchew (2001) described *uruga* as diarrhoea with anorexia, emaciation and death. In this study, *undahi* was described to be a severe diarrhoeal disease, although some people mentioned that lacrimation, nasal discharge and swelling also occurred. Mariner *et al.* (1994) and Gari *et al.* (2015) both suggested that *endahi* or *undahe* could be PPR, with Mariner *et al.* (1994) describing *endahi* as an acute disease with signs of lacrimation, nasal discharge, dyspnoea, coughing, foul-smelling diarrhoea and death after two days, or a chronic disease with weight-loss and death after some time. Gari *et al.* (2015) described *undahe* as a transmissible disease of sheep with signs of diarrhoea, lacrimation, blindness, nasal discharge, head swelling, and emaciation.

**Pox-related syndromes**

As in this study, the earlier studies named *korbuda* (Mariner *et al.* 1994), *korbor* (Dagnatchew 2001) and *korboda* (Gari *et al.* 2015) as causing circular hard skin swellings all over the body, or mainly on the head, udder and under the tail. Mariner *et al.* (1994) also mention signs of lacrimation or nasal discharge, and more severe disease in kids and lambs, causing high mortality. However, none of these authors mentioned the internal *korboda* syndrome described in this study.

**Skin disease**

In the previous studies, the descriptions of *sandera* were similar to the findings of this study; a skin disease with wart-like lesions or thick scabs around the face, perineum, lower leg, and other parts of the body, and their descriptions of *agara* were also similar to the findings of this study (Mariner *et al.* 1994, Dagnatchew 2001, Gari *et al.* 2015). Gari *et al.* (2015) suggested that *agara* was caused by mange mites, and also described *enkata,* which occurred during drought and sucked blood, causing weakness and emaciation. Mariner *et al.* (1994) reported *inkata* to be lice. The previous studies reported *kilim* to be ticks (Mariner *et al.* 1994), which sucked blood and made wounds that became abscesses (Dagnatchew 2001), or caused lameness and *sandera* (Gari *et al.* 2015).

**Lameness**

Mariner *et al.* (1994) described *eiba* to be a foot problem that started in wet conditions, causing lameness and interdigital swelling or abscess, and chronic lameness in some animals, while Dagnatchew (2001) used the term *koss* for lameness in one or more legs that occurred during the rainy season, and Gari *et al.* (2015) described *kos* as lameness caused by tick bites or thorns, leading to swelling and pus.

Similar to *abeb* reported in this study, the previous studies gave descriptions of *abieb* or *abib*, as a disease of cattle, sheep and goats with mouth and interdigital lesions, salivation and lameness (Mariner *et al.* 1994, Dagnatchew 2001, Gari *et al.* 2015).

**Urinary syndromes**

Mariner *et al.* (1994) and Gari *et al.* (2015) described *abali* as a disease of cattle, sheep and goats, causing fever, weakness, anorexia, weight loss and bloody urine, and suggested that the disease was babesiosis. At post mortem, the carcass was yellow and the liver and gall bladder were enlarged. Mariner *et al.* (1994) reported *andero* as a disease of cattle, sheep and goats that was a chronic form of *abali,* with signs of emaciation, diarrhoea and eventually death. At post mortem the carcass was yellow. They suggested this was anaplasmosis. Dagnatchew (2001) described *andera* as affecting goats more than sheep, with signs of diarrhoea, yellow-green eyes and red urine.

**Neurological syndromes**

*Aranwagit* was only reported by Gari *et al.* (2015) who described *arawagit* in goats with signs of raised head, circling, muscle tremor, convulsions and death. In this study, the term *slayti* was associated with non-specific signs and was not clearly characterised but Mariner *et al.* (1994) reported *silayto* as paralysis that was possibly caused by tick bites.

**15. Additional terms used in other studies (not encountered during this study)**

*Adim/Arim: p*us-filled swelling under the skin of goats, around the jaw and pre-scapular lymph node (Gari *et al* 2015).

*Arabah/ Funoyita: b*loat due to ingestion of a certain plant, affecting goats (Gari *et al* 2015).

*Awul: c*ircling, turning neck to the side, falling over, affecting sheep (Gari *et al* 2015).

*Bagidaria: g*astrointestinal worms, affecting sheep (Gari *et al* 2015).

*Dega Meka:* paddling movement in hind legs and the death, affects sheep (Gari *et al* 2015)

*Lubale, duble:* causes oedematous swelling of lower jaw area, especially sheep but also goats, diarrhoea, poor condition, and rough coat. It mainly affects adults. Reported to be bottle jaw due to worms or liver fluke (Mariner et al, 1994). *Dubbele* was described as a disease of sheep that occurs during drought causing oedematous swelling under the neck, head swelling and nasal discharge (Gari *et al* 2015).

*Noke, noki:* fever, rough hair coat, grunting, and congested conjunctiva. Bloody fluid is found in the abdominal cavity (Gari *et al* 2015).

**References**

Dagnatchew, Z. (2001) 'Traditional veterinary practices in Aba'ala Wereda' in OSSREA, ed., Dryland Husbandry in Ethiopia - Research Report Addis Ababa, Ethiopia: OSSREA, 49-74.

Gari, G., Mekonnen, G., Sibhat, D., Ashebir Abebe, A., Sahle, M. and Abie, G. (2015) 'Participatory disease surveillance (PDS) of sheep and goats diseases in selected districts of Afar Regional State: particular focus on peste des petit ruminants (PPR) and sheep and goat pox disease (SGP)', Ethiopian Veterinary Journal, 19(1), 83-105.

Mariner, J. C., Admassu, B., Akabwai, D. and van't Klooster, G. (1994) Lexicon of Afar Disease Terms Middle Awash Region.

1. *sura* means mucus, *san* means nose [↑](#footnote-ref-1)
2. *iba’adu* means “white legs” (*ID20*) [↑](#footnote-ref-2)
3. The rose Bengal test is a spot agglutination test for *Brucella* antibody using stained antigen (OIE (2016) 'Chapter 2.1.4 Brucellosis (Brucella abortus, B. melitensis, and B. suis) (Infection with B. abortus, B.melitensis and B. suis)' in *OIE Terrestrial Manual 2016*, Paris: OIE, 1-44. [↑](#footnote-ref-3)
